# Observation of Near-Field Thermal Radiation between Coplanar Nanodevices with Subwavelength Dimensions

**DOI:** 10.1021/acs.nanolett.3c03748

**Published:** 2024-01-26

**Authors:** Xiao Luo, Hakan Salihoglu, Zexiao Wang, Zhuo Li, Hyeonggyun Kim, Xiu Liu, Jiayu Li, Bowen Yu, Shen Du, Sheng Shen

**Affiliations:** † Department of Mechanical Engineering, Carnegie Mellon University, Pittsburgh, Pennsylvania 15213, United States

**Keywords:** coplanar nanodevices, subwavelength dimensions, near-field thermal radiation, subwavelength confinements, thermal photonic nanodevices

## Abstract

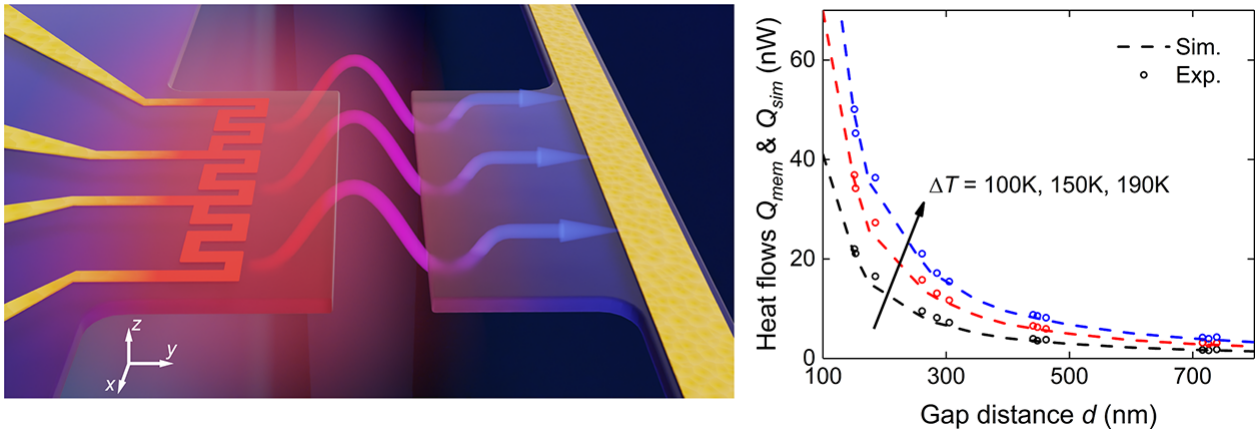

With the continuous advancement of nanotechnology, nanodevices have become crucial components in computing, sensing, and energy conversion applications. The structures of nanodevices typically possess subwavelength dimensions and separations, which pose significant challenges for understanding energy transport phenomena in nanodevices. Here, on the basis of a judiciously designed thermal photonic nanodevice, we report the first measurement of near-field energy transport between two coplanar subwavelength structures over temperature bias up to ∼190 K. Our experimental results demonstrate a 20-fold enhancement in energy transfer beyond blackbody radiation. In contrast with the well-established near-field interactions between two semi-infinite bodies, the subwavelength confinements in nanodevices lead to increased polariton scattering and reduction of supporting photonic modes and, therefore, a lower energy flow at a given separation. Our work unveils exciting opportunities for the rational design of nanodevices, particularly for coplanar near-field energy transport, with important implications for the development of efficient nanodevices for energy harvesting and thermal management.

Nanodevices play a vital role in a broad range of emerging technologies, including quantum computing,^1^ nanophotonic biosensors,^2^ and widely tunable nanolasers.^3^ These devices often consist of active elements with subwavelength dimensions and separations (λ < ∼10 μm at room temperature), which give rise to unusual energy transport paths, such as super-Planckian far-field thermal radiation^4^ and quantum fluctuational energy transport, between the subwavelength elements across vacuum separations via Casimir effect.^5−7^ Particularly at subwavelength separations, near-field thermal radiation exhibits remarkable energy transport enhancements and exceeds well-established blackbody radiation by orders of magnitude.^8−11^ Leveraging such near-field enhancements between nanoscale elements opens up extraordinary applications of nanodevices in thermal management^12,13^ and energy harvesting.^14−16^ However, the realization of near-field-based thermal nanodevices has remained elusive because of the challenges in fabrication and measurement. Previous experimental near-field studies^11,17−35^ have extensively focused on the near-field radiation involving macroscopic structures much larger than wavelengths. Among these studies, near-field thermal signal would be buried within heat conduction for devices based on micropillars/posts/particles support^17−25^ when the dimensions of the emitter and receiver are comparable with the supporting structures. Devices based on nanopositioning platforms^11,26−35^ face the challenge of aligning the subwavelength structures. On-chip devices^8,36^ can circumvent these abovementioned issues but face the difficulty of realizing coplanarity for subwavelength structures due to deflection.^4^ Besides, subwavelength structures also indicate a smaller heat exchange area and, thus, a smaller thermal signal. So far, experiments reporting radiation thermal conductance below 1 nW/K are very limited.^9^ Current knowledge of near-field thermal radiation heavily relies on fluctuational electrodynamic framework derived on the basis of semi-infinite bodies^37,38^ or multilayered structures,^39−41^ which assume infinite dimensions and neglect geometrical constraints of the surfaces. In contrast, subwavelength structures enable distinct energy transport physics in the near field where lateral confinements^42^ may support new guided surface modes or filter certain radiation modes.

To unlock the potential of near-field thermal radiation in nanodevices, here, we employ custom-built coplanar nanodevices that are designed to measure the near-field thermal radiation between two coplanar subwavelength membranes with nanofabricated gaps ranging from ∼150 to ∼750 nm. By integrating them with suspended platinum resistive heating and thermometry, our coplanar nanodevices allow for precise detection of near-field radiative heat transfer between subwavelength surfaces over a wide range of temperature differences up to ∼190 K. Because of the mode suppression from subwavelength confinement, the measured near-field heat flows are observed to be smaller than the analytical predictions derived for the well-known semi-infinite bodies.

As depicted in Figure 1a, our coplanar nanodevice comprises two suspended coplanar silicon nitride membranes with lateral dimensions of 300 nm in thickness (*t*) and 7 μm in length (*l*). The emitting and absorbing membranes are extruded from their corresponding bases by widths (*w*) of 3.5 and 2 μm, respectively. A platinum serpentine heater fabricated on the emitting membrane is used to increase the membrane temperature, whereas a platinum thermometry sensor on the absorbing membrane measures the temperature rise induced by near-field radiative heat transfer. To avoid the influence of heat conduction, the silicon substrate beneath the silicon nitride membranes is etched to form a suspended region (marked in Figure 1b). On the absorbing membrane, a 100 μm long trench is cut behind the platinum to fabricate a 1D long beam structure as the resistive thermometry sensor for heat flow probing. To investigate the coupled near-field interactions between two subwavelength structures (protruded parts on the emitting and absorbing membranes), we fabricate the coplanar nanodevices with a range of nanoscale separations from ∼150 to ∼750 nm using electron beam lithography. As shown in Figure 1c for the tilted-angle scanning electron microscope (SEM) image of a typical nanodevice, the width of the suspended region is controlled to <15 μm to ensure good coplanarity and minimal relative deflection of the membranes. The height profile measurements with a Zyro noncontact profilometer reveal a height deviation of ∼50 nm across the separation gap (Figure 1c, inset). By designing the serpentine heater, we achieve a uniform temperature profile along the *y*-axis within the first 2 μm deep region and minimize the temperature increase at the base of the emitting membrane along the *x*-axis (see the Supporting Information for the simulated temperature profile). We specifically design for a uniform temperature distribution along the first 2 μm from the edge because the near-field radiation is dominated by the first ∼2 μm from the edge (see the Supporting Information). The suspended base efficiently dissipates heat along the *y*-axis because of the short pathway to the intact silicon wafer (as the heat sink), and the temperature drops to ambient temperature within a short distance, which ensures the minimized thermal emission from the base of the emitting membrane to the absorbing membrane. Meanwhile, we also design reference devices to measure any background radiation absorbed by the sensor beam (see the Supporting Information for reference device design).

**Figure 1:**
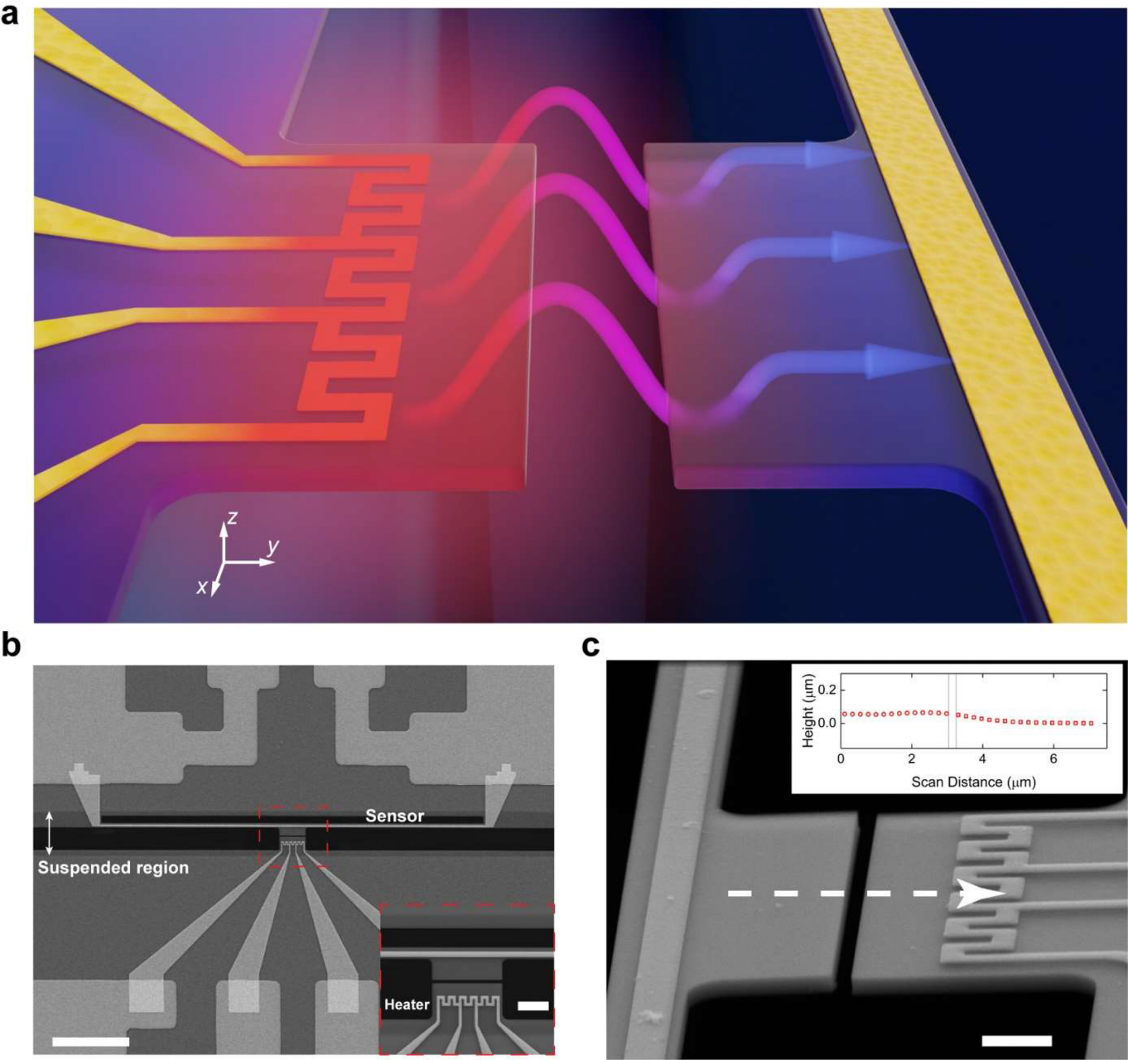
Nanodevice-enabled near-field thermal radiation between parallel subwavelength membranes. (a) Schematic of a near-field thermal nanodevice. The extruded emitting membrane (left) is heated up by platinum serpentine heater, while the extruded absorbing membrane (right) absorbs the near-field radiation with a temperature rise monitored by the platinum resistance thermometer. (b) Top-view SEM images of the measurement nanodevice. Scale bar: 25 μm. The inset shows the zoomed-in SEM image. Scale bar: 3 μm. To physically isolate the emitting and absorbing membranes and, thus, exclude heat conduction, the heater and sensor structures are suspended by etching the underlying silicon substrate, as shown by the suspended region. The width of the suspended region is confined within 15 μm to maintain the coplanarity of the two membranes. (c) Tilted SEM image taken from 70° altitude angle. Scale bar: 1 μm. The inset shows the measured profile along the dashed arrow indicating a height difference of ∼50 nm.

Figure 2a illustrates the measurement scheme and the corresponding thermal circuits in our experiments. All measurements are performed under high vacuum (10^–3^ Pa) with an ambient temperature, *T*_amb_, maintained at 300 K by a temperature controller. Here, the Si substrate acts as a thermal reservoir because of the very large dimensions (∼7 mm long and wide with 500 μm thickness) compared with the nanodevice dimensions and has good thermal contact with the chamber environment maintained at 300 K. Thus, the base temperature of the emitting membrane and the temperatures at both ends of the suspended beam are held at *T*_amb_. A DC current *I* is supplied to the serpentine heater for heating the emitting membrane, and the substrate dissipates the Joule heating with thermal resistance *R*_th,h_. For our near-field thermal measurement, it is not necessary to calibrate the thermal resistance *R*_th,h_. The increase of the heater temperature Δ*T*_h_ (Δ*T*_h_ = *T*_h_ – *T*_amb_) is monitored by supplying a small sinusoidal current (0.4 μA at the 211 Hz frequency) and measuring the corresponding four-wire voltage change, Δ*V*_h_. To measure the temperature variation of the absorbing membrane Δ*T*_s_ (Δ*T*_s_ = *T*_s_ – *T*_amb_), we use a Wheatstone bridge circuit^43^ to detect the voltage change across the long platinum thermometry beam sensor, which dissipates heat to both ends of the sensor via heat conduction by a thermal resistance *R*_th,s_. For the long and thin suspended beam sensor, it is well justified to assume a 1D heat conduction along the sensor beam with the temperatures of its two ends equal to the ambient temperature.^44^ With the thermal radiation input on the absorbing membrane, the corresponding temperature profile along the beam sensor results in the voltage change, Δ*V*_s_ (see the Supporting Information for the temperature profile along the beam sensor). Figure 2b shows the measured Δ*T*_h_ and Δ*T*_s_ for a 150 nm gap nanodevice, while the inset demonstrates the corresponding readings of Δ*V*_h_ and Δ*V*_s_ for the supplied heating current. The corresponding thermal radiation, *Q*_rad_, is determined by *Q*_rad_ = *G*_s_Δ*T*_s_, where *G*_s_ is the heat conductance of the beam sensor to ambient, and *G*_s_ = 1/*R*_th,*s*_. To calibrate *G*_s_, we apply the Joule heating *Q*_s,J_ to the sensor by supplying a DC current with a small sinusoidal current and monitor the corresponding average temperature increase of the sensor  from the four-wire sinusoidal voltage change:  using the 1D heat conduction model (see the Supporting Information for derivations). By knowing *G*_s_ and reading Δ*T*_s_ under a temperature bias Δ*T* (Δ*T* = Δ*T*_h_ – Δ*T*_s_), we measure the thermal radiation heat flow *Q*_rad_. This measurement procedure is repeated for all the nanodevices with various separation gaps and reference devices over a range of ∼190 K temperature bias (Δ*T* = Δ*T*_h_ – Δ*T*_s_ ≈ Δ*T*_h_), i.e., 300–490 K absolute temperature of the heater. To determine the near-field thermal radiation between two extruded membranes, *Q*_mem_, we deduct the background radiation heat flow measured by the reference device *Q*_ref_ from the measured heat flow *Q*_rad_.

**Figure 2:**
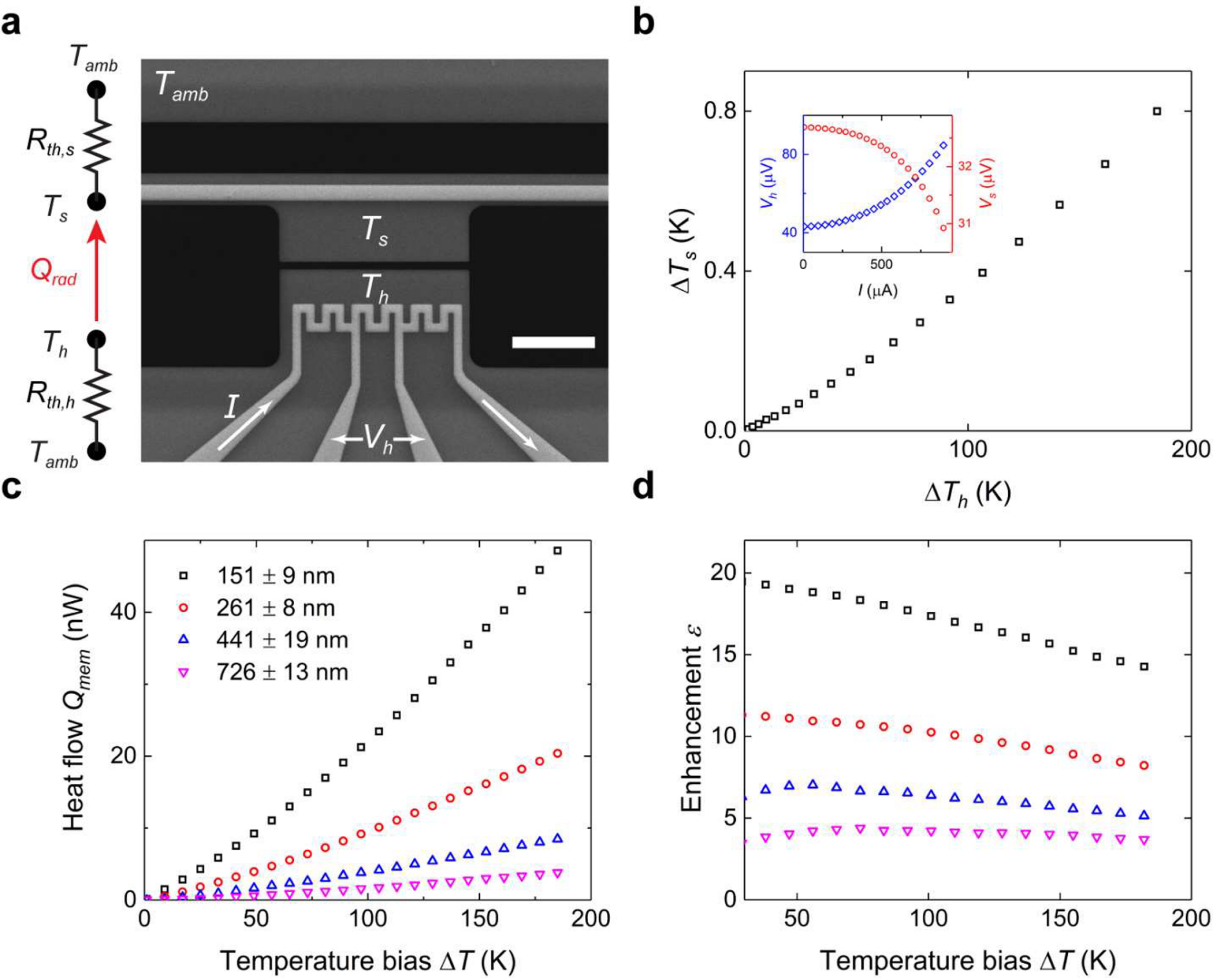
Near-field measurement setup and experimental results. (a) Measurement setup and corresponding thermal circuit. The absorbed radiation heat flow *Q*_rad_ is dissipated to the ambient via heat conduction, which induces a temperature rise of the sensor. Scale bar: 3 μm. (b) Measured temperature increase of the absorbing membrane and corresponding temperature rise of the emitting membrane. The temperature increases of the emitting Δ*T*_h_ and absorbing Δ*T*_s_ membranes are monitored by the four-probe voltage *V*_h_ and gap voltage of the Wheatstone bridge *V*_s_, respectively. The inset shows the measured voltages as a function of heating current, *I*, for a near-field nanodevice with a 151 nm separation. (c) Measured radiation heat flow as a function of temperature bias of different gap distances. For the maximum temperature bias considered, the heat flow increases from ∼4 nW to ∼50 nW as the gap distance decreases from 726 to 151 nm, corresponding to maximum radiation heat conductance of ∼0.26 nW/K. (d) Heat flow enhancement ratio compared with blackbody radiation calculated by *Q*_bb_ = *σAF*_12_(*T*_h_^4^ – *T*_s_^4^).

We measure the radiation heat flows *Q*_mem_ between the extruded membranes in 12 coplanar nanodevices, categorized into four groups on the basis of gap distances. Figure 2c shows the typical *Q*_mem_ as a function of the temperature bias Δ*T* from each group of gap distances in which the corresponding gap distances are measured from SEM images (see the Supporting Information for gap distance measurement). Across all of the nanodevices, we observe that the radiation heat flow *Q*_mem_ increases with an increase in temperature bias. Furthermore, at a given temperature bias, *Q*_mem_ rises with reduced gap separation because of the near-field effect. For instance, when the maximum temperature bias of 190 K is applied in our experiments, reduction of the gap distance from 726 to 151 nm leads to a significant heat flow increase from ∼4 to ∼50 nW, and the corresponding radiation thermal conductance *G*_mem_ increases from ∼0.02 to ∼0.26 nW/K. This behavior confirms the enhanced near-field thermal radiation between the membranes at smaller gap distances.

To quantify the near-field enhancement of the membranes, we define an enhancement ratio ε = *Q*_mem_/*Q*_bb_. Here, *Q*_bb_ = *σAF*_12_(*T*_h_^4^ – *T*_s_^4^) is the radiation heat flow between two blackbodies with the same configuration as the coplanar nanodevice, where σ is the Stefan–Boltzmann constant, *A* is the exchange surface area, and *F*_12_ is the view factor between the exchange surfaces. We plot the enhancement ratio ε as a function of the temperature bias in Figure 2d. Interestingly, over the 190 K temperature bias range, we observe that ε decreases as the temperature bias increases, which will be further discussed in the simulated thermal radiation spectrum. As gap distance increases from 151 to 726 nm, the maximum radiation heat flow decreases by 92% (∼50 to ∼4 nW), while the maximum enhancement ratio (ε) only decreases by 80% (∼20 to ∼4) because the view factor *F*_12_ also drops significantly from ∼0.6 to ∼0.2 (see the Supporting Information for the calculation of view factor). The maximum enhancement ratio that we measured is ∼20 at a 30 K temperature bias and ∼150 nm gap distance. We notice that the enhancement ratio is less than the 100-fold enhancement reported in the far field with a similar membrane thickness.^4^ We believe that such a discrepancy arises because blackbody radiation also depends on gap distance for coplanar membranes. Blackbody radiation is much weaker at a far-field gap distance than that at a near-field gap distance (see the Supporting Information for the calculations and discussions).

To validate our near-field measurements, we conduct fluctuational electromagnetic (fluctuational-EM) simulation using the fluctuational surface current (FSC) method,^45,46^ which considers the two coplanar silicon nitride membranes with the design dimensions of 7 μm × 2 μm × 0.3 μm. Figure 3a shows the surface mesh used for the FSC method, on the basis of which the reduced heat flow spectra *S*(ω) are calculated. From this, the simulated near-field heat flow *Q*_*sim*_ can be determined by
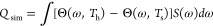
1where Θ(ω, *T*) = ℏω/[exp(ℏω/*k*_B_*T*) – 1] represents the Planck energy per oscillator at the temperature *T*. In Figure 3b, we compare the simulation results to the measurements from the four devices in Figure 2c as a function of temperature bias. For all 12 measured devices, we further compare the simulation results with their measured radiation heat flows as a function of gap distance at multiple temperature biases in Figure 3c. In both temperature- and gap-dependent comparisons, the measured heat flows exhibit good agreement with the simulation results for all the measured separation gaps, thus further validating our experimental results. To elaborate on the mechanism of near-field enhancement, we plot the reduced heat flow spectra of different gap distances in Figure 3d. The major peak around 1.8 × 10^14^ rad/s corresponds to the resonance of surface phonon polaritons supported by the silicon nitride membrane. In Figure 3d, the reduced heat flow dramatically increases with a decreased gap distance, thereby indicating the strong near-field effect. While the resonance frequency of surface phonon polaritons remains fixed with temperature, the radiative thermal energy peak (based on Wien’s law) shifts to higher frequencies as temperature increases, which results in *Q*_bb_ increasing with a rate higher than that of *Q*_mem_ for the same Δ*T*. Consequently, the near-field enhancement ε decreases with increasing Δ*T* values, as shown in Figure 2d.

**Figure 3:**
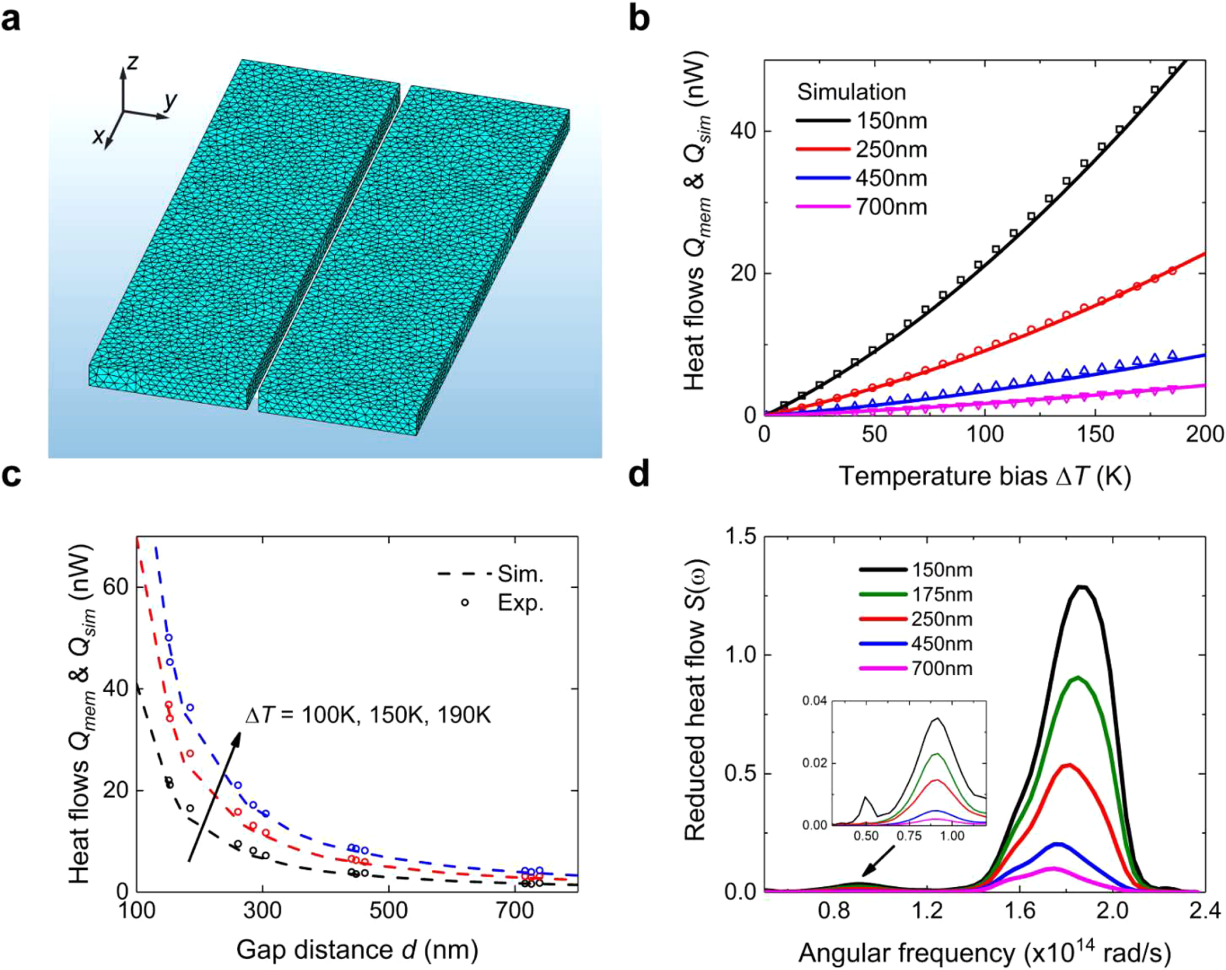
Fluctuational-EM simulation and comparison with measurements. (a) Surface mesh generated for the FSC method. (b) Comparison between the simulated and measured heat flows. The measurements show excellent agreement with the simulations. (c) Comparison between the simulated and measured heat flows as a function of gap distance for the temperature biases of 100, 150, and 190 K, respectively. (d) Reduced heat flow spectra. The inset shows the zoomed-in spectrum in the low frequency range.

To elucidate the impact of subwavelength dimensions in near-field radiative heat transfer, we compare our simulation results for the subwavelength surfaces with the analytical predictions based on the near-field heat flux between two semi-infinite bodies. Figure 4a illustrates that the subwavelength surfaces (solid lines) exchange less heat than the semi-infinite bodies (dashed lines) for the same exchange area of *l* × *t* = 7 × 0.3 μm^2^. The observed lower heat flow arises from the boundary scattering of surface phonon polaritons due to the presence of the subwavelength structures, in which some scattered waves escape from the gap and do not contribute to the near-field heat transfer.

**Figure 4:**
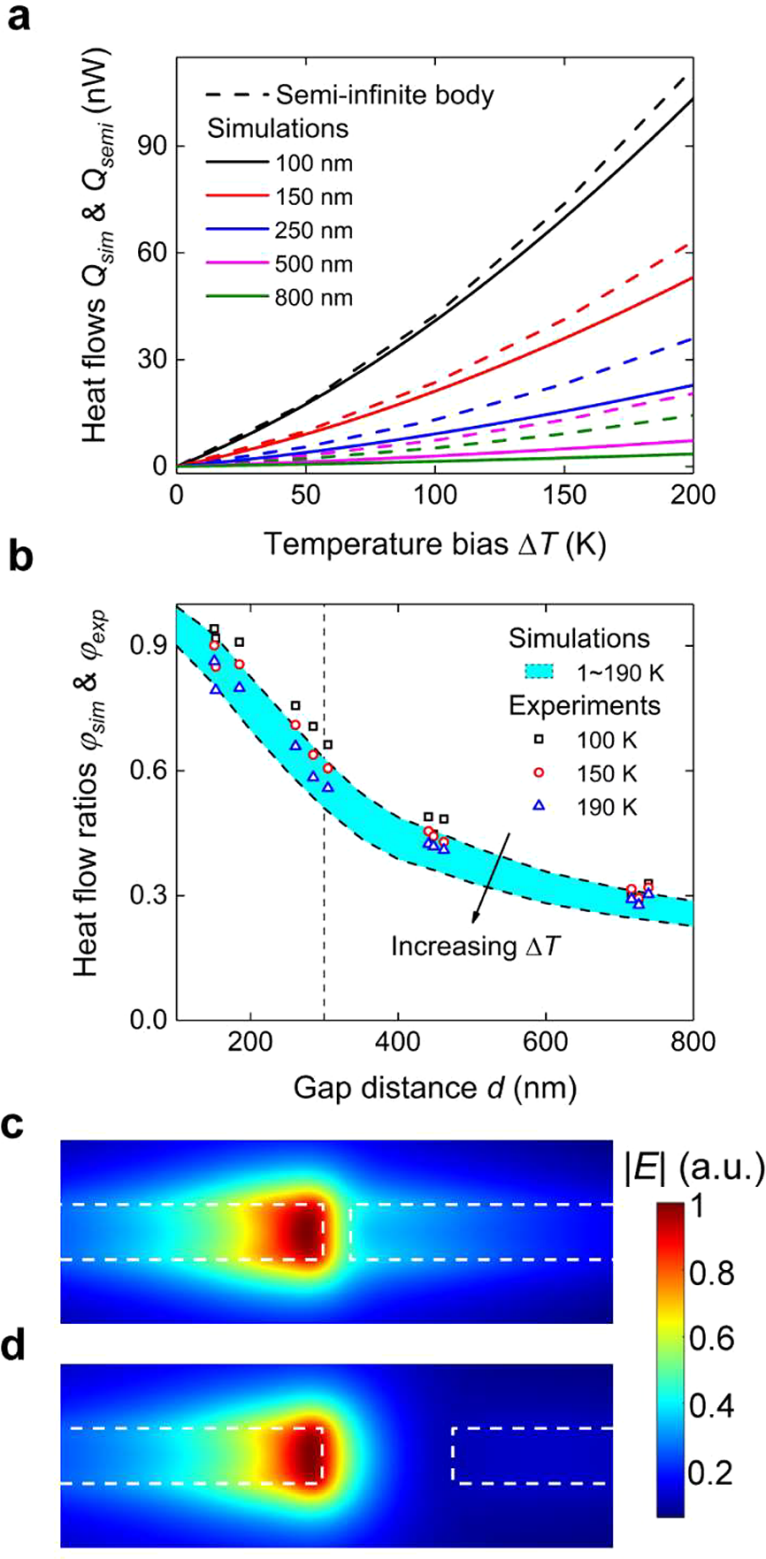
Theoretical analysis by comparing with the semi-infinite body theory. (a) Comparison between the heat flows from semi-infinite body theory (dashed line) and fluctuational-EM simulation (solid line). The near-field radiation heat transfer between the subwavelength membranes is lower than the prediction between semi-infinite bodies. We attribute the lower heat flow across the subwavelength surfaces to the energy carried by the polaritons scattered away from the gap. (b) Heat flow ratio φ_sim_ (shaded region) and φ_exp_ (dots) as a function of gap distance where φ_sim_ and φ_exp_ are the ratios of fluctuational-EM simulation and experimental measurements to semi-infinite body predictions, respectively. φ_sim_ or φ_exp_ dramatically increases from ∼20% to almost 100% as the gap distance decreases from 800 to 100 nm. Besides, φ_sim_ or φ_exp_ increases with a higher rate as the gap distance decreases below the thickness, 300 nm. (c,d) Electric field profiles between the parallel membranes with gap distances of 150 and 700 nm, respectively. The electric field profile at the 150 nm gap distance is more uniformly distributed within the separation.

In Figure 4b, we define and plot a heat flow ratio, φ_sim_ = *Q*_sim_/*Q*_semi_ with respect to separation gap within the temperature range of interest, where *Q*_semi_ is the predicted heat flow, by multiplying the near-field heat flux between two semi-infinite bodies with the same cross section area (*l* × *t*). As a comparison, we also introduce the other heat flow ratio, φ_exp_ = *Q*_mem_/*Q*_semi_, which shows excellent agreement with φ_sim_ in Figure 4b. At large separations (*d* > 500 nm), the difference between *Q*_sim_ (or *Q*_exp_) and *Q*_semi_ is significant (e.g., φ_sim_ = ∼0.2 at *d* = 800 nm). However, as the separation decreases, the heat transfer rate between the subwavelength surfaces increases and approaches that between the semi-infinite bodies (e.g., φ_sim_ > 0.5 for *d* < 300 nm). The increase in φ_sim_ (or φ_exp_) depends on two main criteria. First, at large separations, the surface phonon polaritons have wavelengths, λ, that are larger than but comparable with *t*. At shorter separations, the surface phonon polaritons possess smaller wavelengths and interact less with the lateral *x* and *z* dimensions (see the Supporting Information for polariton and wavelength relations). As a result, the scattering of these polaritons at shorter gap distances is less pronounced compared with that at larger separations. Figure 4c,d shows the electric field profile around the hot region for separation gaps of 150 and 700 nm, respectively. As seen, the field away from the surface (light blue and cyan regions) is spatially distributed along the thickness because of scattering. However, the field closer to the surface (yellow and green regions) is more uniformly distributed, thereby resembling the semi-infinite bodies (no lateral dependence of the field profile). This behavior leads to the heat transfer rate closer to that between the semi-infinite bodies at shorter distances. Second, the rate of the increase in φ_sim_ (or φ_exp_) is enhanced for the separations below the thickness of the membrane. Spatial confinement along the *z*-axis prevents the existence of polaritons with wavelengths larger than thickness *t*. These polaritons are only supported along the length direction (*x*-axis) and become the main contributors to energy transport at large separations. At separations less than the thickness *t*, polaritons with wavelength smaller than thickness mainly transport energy across the gap, and these polaritons exist along both the thickness (*z*-axis) and the length (*x*-axis) directions. Thus, at the short separations, the contributing polaritons supported in both directions carry a higher total energy and approach the semi-infinite bodies where polaritons exist in all directions without any spatial constraint.

In this work, we design and fabricate coplanar nanodevices to measure the near-field thermal radiation between subwavelength coplanar membranes. Through high-sensitivity measurements, we accurately quantify the near-field heat flow, which shows excellent agreement with fluctuational electrodynamics simulations. Compared with blackbody radiation, we observe 20-fold enhancement in thermal radiation between the two subwavelength structures with a separation gap of ∼150 nm, while the maximum thermal conductance is still much less than these previously reported in near-field experiments because of the small heat exchange area of the subwavelength structure. In addition, our analysis finds that the near-field thermal radiation between the subwavelength coplanar membranes remains lower than that based on the semi-infinite model, which is attributed to energy escaping from the subwavelength confinements due to scattering of polaritons and less contributions from polariton modes with large wavelengths. Our findings elucidate the near-field energy transfer between subwavelength structures, thereby paving the way for the development of coplanar near-field nanodevices for energy harvesting and thermal management.

## Supporting Information Available

Fabrication process, gap distances measurements of the devices, temperature profile on the emitting membrane, near-field thermal emission from the emitting membrane, reference devices, overview of thermal measurement, temperature-resistance calibration of PRT, thermal frequency domain responses of the heater and sensor, determining temperature variations from the platinum thermometer, temperature profile along the sensor beam and calibration of *G*_s_, noise floor of the measurement, linear interpolations of raw data, radiation heat flows of all measured devices, radiation heat conductance of all measured devices, near-field enhancements of all measured devices, view factor calculation, dielectric permittivity of silicon nitride, comparison with far-field super-Planckian thermal radiation, reflection measurement of the silicon nitride membrane, semi-infinite body calculation, polariton and wavelength relation, electric field simulation, and the influence of height deviation (PDF)

## Notes

During the preparation of this manuscript, S.S. and X.Luo learned that the group of Prof. Chris Dames at UC Berkeley is also preparing a manuscript (arXiv preprint arXiv:2310.20356) on lateral near-field thermal radiation between silicon carbide membranes.

## References

[ref1] Heinrich A. J.; Oliver W. D.; Vandersypen L. M. K.; Ardavan A.; Sessoli R.; Loss D.; Jayich A. B.; Fernandez-Rossier J.; Laucht A.; Morello A. Quantum-Coherent Nanoscience. Nat. Nanotechnol. 2021, 16 (12), 1318–1329. 10.1038/s41565-021-00994-1. 34845333

[ref2] Altug H.; Oh S.-H.; Maier S. A.; Homola J. Advances and Applications of Nanophotonic Biosensors. Nat. Nanotechnol. 2022, 17 (1), 5–16. 10.1038/s41565-021-01045-5. 35046571

[ref3] Corato-Zanarella M.; Gil-Molina A.; Ji X.; Shin M. C.; Mohanty A.; Lipson M. Widely Tunable and Narrow-Linewidth Chip-Scale Lasers from near-Ultraviolet to near-Infrared Wavelengths. Nat. Photonics 2023, 17 (2), 157–164. 10.1038/s41566-022-01120-w.

[ref4] Thompson D.; Zhu L.; Mittapally R.; Sadat S.; Xing Z.; McArdle P.; Qazilbash M. M.; Reddy P.; Meyhofer E. Hundred-Fold Enhancement in Far-Field Radiative Heat Transfer over the Blackbody Limit. Nature 2018, 561 (7722), 216–221. 10.1038/s41586-018-0480-9. 30177825

[ref5] Rodriguez A. W.; Capasso F.; Johnson S. G. The Casimir Effect in Microstructured Geometries. Nat. Photonics 2011, 5 (4), 211–221. 10.1038/nphoton.2011.39.

[ref6] Fong K. Y.; Li H.-K.; Zhao R.; Yang S.; Wang Y.; Zhang X. Phonon Heat Transfer across a Vacuum through Quantum Fluctuations. Nature 2019, 576, 243–247. 10.1038/s41586-019-1800-4. 31827291

[ref7] Xu Z.; Gao X.; Bang J.; Jacob Z.; Li T. Non-Reciprocal Energy Transfer through the Casimir Effect. Nat. Nanotechnol. 2022, 17 (2), 148–152. 10.1038/s41565-021-01026-8. 34903895

[ref8] St-Gelais R.; Zhu L.; Fan S.; Lipson M. Near-Field Radiative Heat Transfer between Parallel Structures in the Deep Subwavelength Regime. Nat. Nanotechnol. 2016, 11 (6), 515–519. 10.1038/nnano.2016.20. 26950243

[ref9] Lucchesi C.; Vaillon R.; Chapuis P.-O. Radiative Heat Transfer at the Nanoscale: Experimental Trends and Challenges. Nanoscale Horizons 2021, 6 (3), 201–208. 10.1039/D0NH00609B. 33533775

[ref10] Song B.; Fiorino A.; Meyhofer E.; Reddy P. Near-Field Radiative Thermal Transport: From Theory to Experiment. AIP Adv. 2015, 5 (5), 053503 10.1063/1.4919048.

[ref11] Shen S.; Narayanaswamy A.; Chen G. Surface Phonon Polaritons Mediated Energy Transfer between Nanoscale Gaps. Nano Lett. 2009, 9 (8), 2909–2913. 10.1021/nl901208v. 19719110

[ref12] Zhu L.; Fiorino A.; Thompson D.; Mittapally R.; Meyhofer E.; Reddy P. Near-Field Photonic Cooling through Control of the Chemical Potential of Photons. Nature 2019, 566 (7743), 239–244. 10.1038/s41586-019-0918-8. 30760913

[ref13] Latella I.; Biehs S.-A.; Ben-Abdallah P. Smart Thermal Management with Near-Field Thermal Radiation. Opt. Express 2021, 29 (16), 24816 10.1364/OE.433539. 34614829

[ref14] Mittapally R.; Lee B.; Zhu L.; Reihani A.; Lim J. W.; Fan D.; Forrest S. R.; Reddy P.; Meyhofer E. Near-Field Thermophotovoltaics for Efficient Heat to Electricity Conversion at High Power Density. Nat. Commun. 2021, 12, 4364 10.1038/s41467-021-24587-7. 34272361 PMC8285488

[ref15] Lucchesi C.; Cakiroglu D.; Perez J.-P.; Taliercio T.; Tournié E.; Chapuis P.-O.; Vaillon R. Near-Field Thermophotovoltaic Conversion with High Electrical Power Density and Cell Efficiency above 14%. Nano Lett. 2021, 21 (11), 4524–4529. 10.1021/acs.nanolett.0c04847. 34037401

[ref16] Bhatt G. R.; Zhao B.; Roberts S.; Datta I.; Mohanty A.; Lin T.; Hartmann J.-M.; St-Gelais R.; Fan S.; Lipson M. Integrated Near-Field Thermo-Photovoltaics for Heat Recycling. Nat. Commun. 2020, 11, 2545 10.1038/s41467-020-16197-6. 32439917 PMC7242323

[ref17] Hu L.; Narayanaswamy A.; Chen X.; Chen G. Near-Field Thermal Radiation between Two Closely Spaced Glass Plates Exceeding Planck’s Blackbody Radiation Law. Appl. Phys. Lett. 2008, 92 (13), 133106 10.1063/1.2905286.

[ref18] Ying X.; Sabbaghi P.; Sluder N.; Wang L. Super-Planckian Radiative Heat Transfer between Macroscale Surfaces with Vacuum Gaps down to 190 Nm Directly Created by SU-8 Posts and Characterized by Capacitance Method. ACS Photonics 2020, 7 (1), 190–196. 10.1021/acsphotonics.9b01360.

[ref19] Tang L.; Desutter J.; Francoeur M. Near-Field Radiative Heat Transfer between Dissimilar Materials Mediated by Coupled Surface Phonon- And Plasmon-Polaritons. ACS Photonics 2020, 7 (5), 1304–1311. 10.1021/acsphotonics.0c00404.

[ref20] Sabbaghi P.; Long L.; Ying X.; Lambert L.; Taylor S.; Messner C.; Wang L. Super-Planckian Radiative Heat Transfer between Macroscale Metallic Surfaces Due to near-Field and Thin-Film Effects. J. Appl. Phys. 2020, 128 (2), 025305 10.1063/5.0008259.

[ref21] Yang J.; Du W.; Su Y.; Fu Y.; Gong S.; He S.; Ma Y. Observing of the Super-Planckian near-Field Thermal Radiation between Graphene Sheets. Nat. Commun. 2018, 9, 4033 10.1038/s41467-018-06163-8. 30279411 PMC6168489

[ref22] DeSutter J.; Tang L.; Francoeur M. A Near-Field Radiative Heat Transfer Device. Nat. Nanotechnol. 2019, 14 (8), 751–755. 10.1038/s41565-019-0483-1. 31263192

[ref23] Lim M.; Song J.; Lee S. S.; Lee J.; Lee B. J. Surface-Plasmon-Enhanced Near-Field Radiative Heat Transfer between Planar Surfaces with a Thin-Film Plasmonic Coupler. Phys. Rev. Appl. 2020, 14, 014070 10.1103/PhysRevApplied.14.014070.

[ref24] Shi K.; Chen Z.; Xing Y.; Yang J.; Xu X.; Evans J.-S.; He S. Near-Field Radiative Heat Transfer Modulation with an Ultrahigh Dynamic Range through Mode Mismatching. Nano Lett. 2022, 22 (19), 7753–7760. 10.1021/acs.nanolett.2c01286. 36162118 PMC9562469

[ref25] Shi K.; Chen Z.; Xu X.; Evans J.-S.; He S. Optimized Colossal Near-Field Thermal Radiation Enabled by Manipulating Coupled Plasmon Polariton Geometry. Adv. Mater. 2021, 33, 2106097 10.1002/adma.202106097. PMC11468567 34632648

[ref26] Lucchesi C.; Vaillon R.; Chapuis P.-O. Temperature Dependence of Near-Field Radiative Heat Transfer above Room Temperature. Mater. Today Phys. 2021, 21, 100562 10.1016/j.mtphys.2021.100562.

[ref27] Bernardi M. P.; Milovich D.; Francoeur M. Radiative Heat Transfer Exceeding the Blackbody Limit between Macroscale Planar Surfaces Separated by a Nanosize Vacuum Gap. Nat. Commun. 2016, 7, 12900 10.1038/ncomms12900. 27682992 PMC5056409

[ref28] Ghashami M.; Geng H.; Kim T.; Iacopino N.; Cho S. K.; Park K. Precision Measurement of Phonon-Polaritonic Near-Field Energy Transfer between Macroscale Planar Structures under Large Thermal Gradients. Phys. Rev. Lett. 2018, 120 (17), 175901 10.1103/PhysRevLett.120.175901. 29756825

[ref29] Giroux M.; Stephan M.; Brazeau M.; Molesky S.; Rodriguez A. W.; Krich J. J.; Hinzer K.; St-Gelais R. Measurement of Near-Field Radiative Heat Transfer at Deep Sub-Wavelength Distances Using Nanomechanical Resonators. Nano Lett. 2023, 23 (18), 8490–8497. 10.1021/acs.nanolett.3c02049. 37671916

[ref30] Lim M.; Song J.; Lee S. S.; Lee B. J. Tailoring Near-Field Thermal Radiation between Metallo-Dielectric Multilayers Using Coupled Surface Plasmon Polaritons. Nat. Commun. 2018, 9 (1), 4302 10.1038/s41467-018-06795-w. 30327494 PMC6191454

[ref31] Song B.; Thompson D.; Fiorino A.; Ganjeh Y.; Reddy P.; Meyhofer E. Radiative Heat Conductances between Dielectric and Metallic Parallel Plates with Nanoscale Gaps. Nat. Nanotechnol. 2016, 11 (6), 509–514. 10.1038/nnano.2016.17. 26950244

[ref32] Song B.; Ganjeh Y.; Sadat S.; Thompson D.; Fiorino A.; Fernández-Hurtado V.; Feist J.; Garcia-Vidal F. J.; Cuevas J. C.; Reddy P.; Meyhofer E. Enhancement of Near-Field Radiative Heat Transfer Using Polar Dielectric Thin Films. Nat. Nanotechnol. 2015, 10 (3), 253–258. 10.1038/nnano.2015.6. 25705866

[ref33] Fiorino A.; Thompson D.; Zhu L.; Song B.; Reddy P.; Meyhofer E. Giant Enhancement in Radiative Heat Transfer in Sub-30 Nm Gaps of Plane Parallel Surfaces. Nano Lett. 2018, 18 (6), 3711–3715. 10.1021/acs.nanolett.8b00846. 29701988

[ref34] Shi K.; Sun Y.; Chen Z.; He N.; Bao F.; Evans J.; He S. Colossal Enhancement of Near-Field Thermal Radiation across Hundreds of Nanometers between Millimeter-Scale Plates through Surface Plasmon and Phonon Polaritons Coupling. Nano Lett. 2019, 19 (11), 8082–8088. 10.1021/acs.nanolett.9b03269. 31646871

[ref35] Salihoglu H.; Nam W.; Traverso L.; Segovia M.; Venuthurumilli P. K.; Liu W.; Wei Y.; Li W.; Xu X. Near-Field Thermal Radiation between Two Plates with Sub-10 Nm Vacuum Separation. Nano Lett. 2020, 20 (8), 6091–6096. 10.1021/acs.nanolett.0c02137. 32628493

[ref36] St-Gelais R.; Guha B.; Zhu L.; Fan S.; Lipson M. Demonstration of Strong Near-Field Radiative Heat Transfer between Integrated Nanostructures. Nano Lett. 2014, 14 (12), 6971–6975. 10.1021/nl503236k. 25420115

[ref37] Loomis J. J.; Maris H. J. Theory of Heat Transfer by Evanescent Electromagnetic Waves. Phys. Rev. B 1994, 50 (24), 18517–18524. 10.1103/PhysRevB.50.18517. 9976287

[ref38] Joulain K.; Mulet J.-P.; Marquier F.; Carminati R.; Greffet J.-J. Surface Electromagnetic Waves Thermally Excited: Radiative Heat Transfer, Coherence Properties and Casimir Forces Revisited in the Near Field. Surf. Sci. Rep. 2005, 57 (3–4), 59–112. 10.1016/j.surfrep.2004.12.002.

[ref39] Basu S.; Yang Y.; Wang L. Near-Field Radiative Heat Transfer between Metamaterials Coated with Silicon Carbide Thin Films. Appl. Phys. Lett. 2015, 106 (3), 033106 10.1063/1.4906530.

[ref40] Boriskina S. V.; Tong J. K.; Huang Y.; Zhou J.; Chiloyan V.; Chen G. Enhancement and Tunability of Near-Field Radiative Heat Transfer Mediated by Surface Plasmon Polaritons in Thin Plasmonic Films. Photonics 2015, 2 (2), 659–683. 10.3390/photonics2020659.

[ref41] Francoeur M.; Mengüç M. P.; Vaillon R. Near-Field Radiative Heat Transfer Enhancement via Surface Phonon Polaritons Coupling in Thin Films. Appl. Phys. Lett. 2008, 93 (4), 043109 10.1063/1.2963195.

[ref42] Biehs S.-A.; Rousseau E.; Greffet J.-J. Mesoscopic Description of Radiative Heat Transfer at the Nanoscale. Phys. Rev. Lett. 2010, 105 (23), 234301 10.1103/PhysRevLett.105.234301. 21231469

[ref43] Wingert M. C.; Chen Z. C. Y.; Kwon S.; Xiang J.; Chen R. Ultra-Sensitive Thermal Conductance Measurement of One-Dimensional Nanostructures Enhanced by Differential Bridge. Rev. Sci. Instrum. 2012, 83 (2), 024901 10.1063/1.3681255. 22380117

[ref44] Zheng J.; Wingert M. C.; Dechaumphai E.; Chen R. Sub-picowatt/kelvin resistive thermometry for probing nanoscale thermal transport. Rev. Sci. Instrum. 2013, 84 (11), 114901 10.1063/1.4826493. 24289425

[ref45] Reid M. T. H.; Johnson S. G. Efficient Computation of Power, Force, and Torque in BEM Scattering Calculations. IEEE Trans. Antennas Propag. 2015, 63 (8), 3588–3598. 10.1109/TAP.2015.2438393.

[ref46] Rodriguez A. W.; Reid M. T. H.; Johnson S. G. Fluctuating-Surface-Current Formulation of Radiative Heat Transfer: Theory and Applications. Phys. Rev. B 2013, 88 (5), 054305 10.1103/PhysRevB.88.054305.

