# Supplementary material for: Observation of Near-Field Thermal Radiation between Coplanar Nanodevices with Subwavelength Dimensions
Source: Nano Lett. 2024 Jan 26;24(5):1502–9. doi: 10.1021/acs.nanolett.3c03748 (PMC10853966; doi:10.1021/acs.nanolett.3c03748)
Supplement: Supplementary file 1 — nl3c03748_si_001.pdf [file nl3c03748_si_001.pdf]

Supplementary Information for

Observation of Near-Field Thermal Radiation between Co-Planar Nanodevices with Sub-Wavelength Dimensions

Xiao Luo,[1†] Hakan Salihoglu,[1†] Zexiao Wang,[1†] Zhuo Li,[1†] Hyeonggyun Kim,[1] Xiu Liu,[1] Jiayu Li,[1] Bowen Yu,[1] Shen Du,[1] Sheng Shen,[1*]

[1]Department of Mechanical Engineering, Carnegie Mellon University, Pittsburgh, PA 15213, USA.

[†]The authors contributed equally.

[*]Corresponding author: Sheng Shen, email: sshen1@cmu.edu

1 Fabrication process

Figure S1 shows the fabrication process of the near-field nanodevice. The fabrication process starts with the deposition of 300 nm thick low-stress silicon nitride films on both sides of a silicon substrate via low-pressure chemical vapor deposition. A 30-nm-thick Pt layer for the heater and sensor structures is then deposited by sputtering and patterned through electron-beam lithography and lift-off processes. A 35-nm Pt electrode is subsequently patterned and deposited using another lift-off process. To create the through-hole structure for heat insulation, a silicon-nitride hard mask is formed on the backside of the chip via reactive ion etching. After that, potassium hydroxide (KOH) wet etching is applied to etch most of the silicon beneath the metal structure. The heater and sensor structures of the silicon nitride are formed by electron-beam lithography and reactive ion etching, followed by another KOH wet etching process to suspend the structure. Finally, critical point drying is performed to release the suspended structure. In total there are 12 devices among 3 batches fabricated. Within each batch there is a reference device.

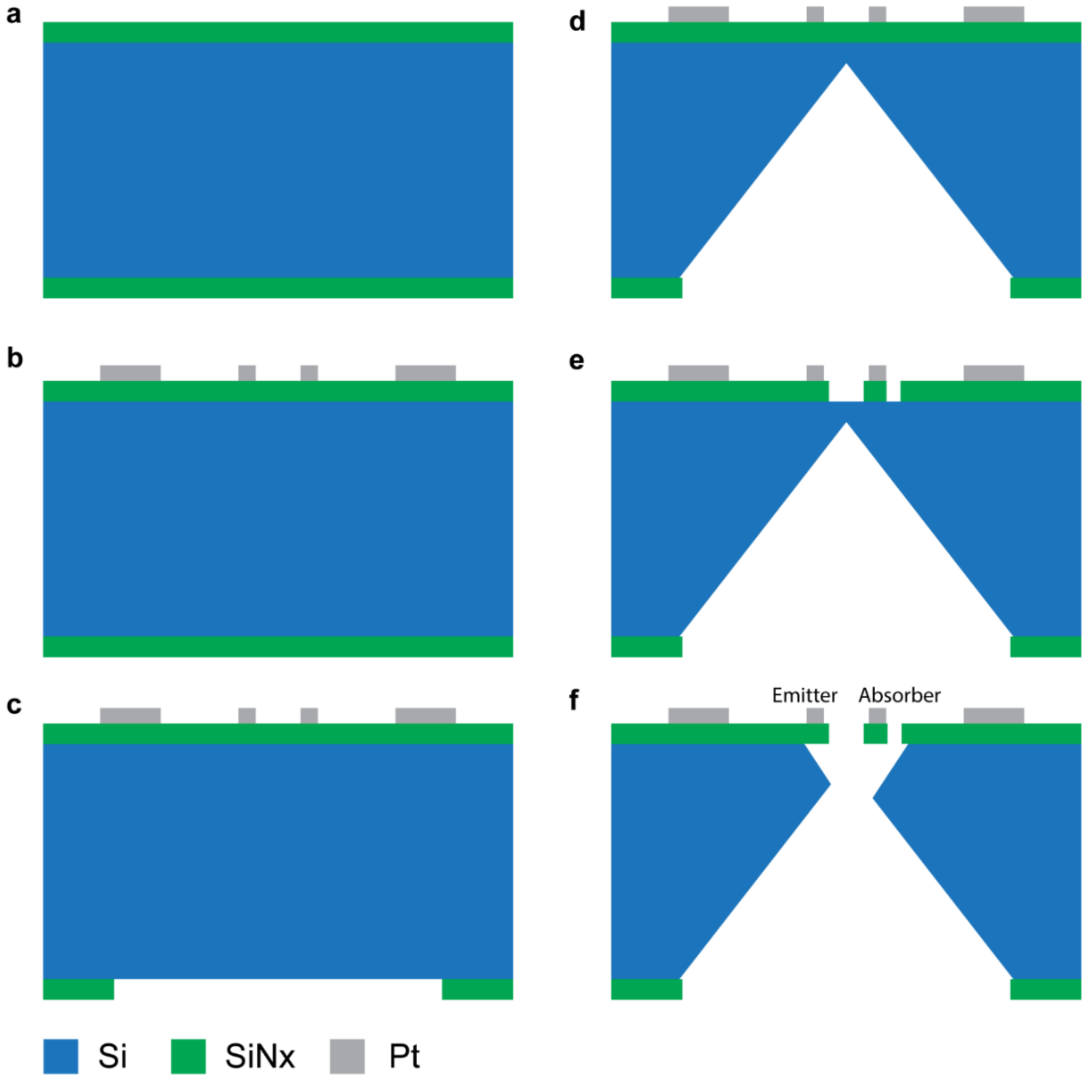

Fig. S1. Fabrication process flow of the suspended nanodevices. a, Deposition of low-stress silicon nitride on both sides of the substrate by low-pressure chemical vapor deposition. **b**, Patterning and deposition of the platinum heater, sensor and electrodes. **c**, Patterning and etching of silicon nitride on the

backside. **d**, KOH wet etching of silicon. **e**, Patterning and etching of silicon nitride on the frontside. **f**, Second KOH wet etching of silicon and critical point drying.

2 Gap distances measurements of the devices

To measure the gap distance distances, we take at least three top-view SEM images for each device with different magnifications (~10000X to ~70000X), and measure gap the distance by measuring the distance between the edges using a data visualization software Gwyddion. The mean value and standard deviation are used as gap distances and errors, respectively. For example, as shown in Fig. S2, the measured gap distances in the three figures are 421 nm, 459 nm and 443 nm, respectively. The gap distance is 441±19 nm. The gap distances of all measured devices are listed in Table S1.

Table S1. Gap distances of all tested devices. * indicates the device whose results are presented in Fig. 2 and Fig. 3. Measured radiation heat flows of all devices are available in the supplementary materials.

Batch	Device	Gap Distance (nm)
A	#1	153±2
	#2*	151±9
	#3*	261±8
	#4	2133±5 (ref)
B	#1	185±4
	#2	285±7
	#3	305±7
	#4	2285±6 (ref)
C	#1*	441±19
	#2	462±18
	#3	448±32
	#4*	726±20
	#5	716±11
	#6	739±25
	#7	2467±9 (ref)

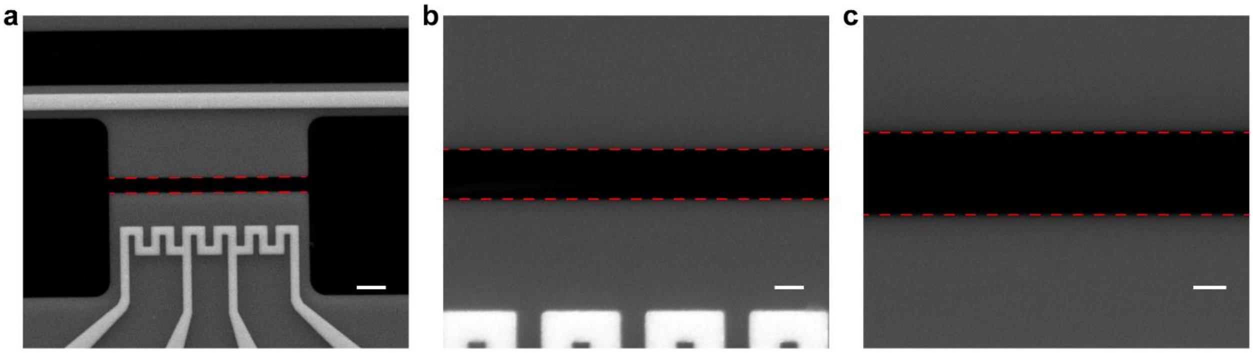

Fig. S2. Top-view SEM images with different magnifications for gap distance measurement. The values of scale bars from left to right: 1 μm **(a)**, 300 nm **(b)** and 200 nm **(c)**.

3 Temperature profile on the emitting membrane

Figure S3 shows the temperature profile on the emitting membrane. Uniform temperature profile is maintained within the first 2-μm of the emitting membrane.

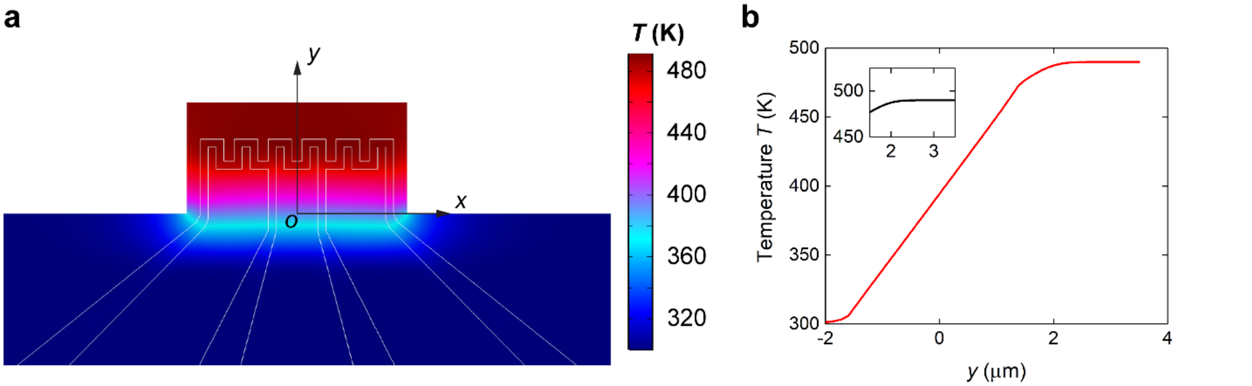

Fig. S3. Simulated temperature profile on the emitting membrane. a, Top-view temperature profile of the emitting membrane. Uniform temperature is observed within the first 2-μm of the emitting membrane with the optimized heater structure. The temperature greatly drops beyond the first 2 μm such that the emission from the base is minimized. **b**, Temperature profile on the emitting membrane along the *y* axis. Inset: Temperature profile within the first 2-μm of the emitting membrane.

4 Near-field thermal emission from the emitting membrane

To investigate the width of the emitting membrane contributing to the near-field thermal radiation, we split the emitting membrane into multiple 7 μm × 0.3 μm × 0.3 μm slots positioned along the width direction (*y* axis) and use the Wiener Chaos expansion (WCE) method[S1] to investigate the contribution from each slot to the near-field radiation, by individually exciting each of them, as shown in Fig. S4a. Figure S4b shows the reduced heat flow (with arbitrary unit) spectra of the ten slots from the edge for a gap distance of 150

nm. The magnitude of the peak in the reduced heat flow drops quickly as the position of the slots gets far from the edge, especially for slots behind slot 7, which corresponds to a distance of ~ 2 μm from the gap.

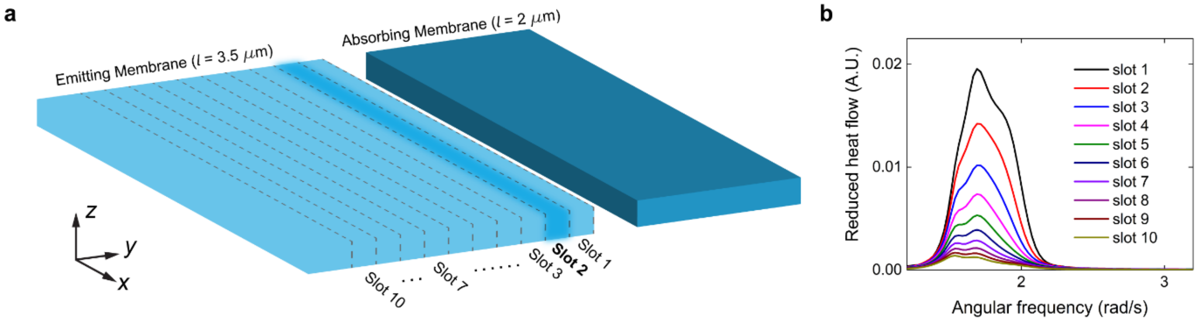

Fig. S4. Near-field thermal emission from the emitting membrane. a, Schematic of the slot-by-slot Wiener Chaos expansion (WCE) simulation. Each slot, e.g., slot 2 in the schematic, is excited individually. **b**, Reduced heat flow spectra of the ten slots from the edge.

5 Reference devices

The reference devices closely resemble the devices for near-field thermal measurements. The only difference is that it has a single beam sensor without the absorbing membrane. Figures. S5 a & b show the SEM images of a reference device. The purpose of the reference device is to measure any parasitic radiation absorbed by the remaining sensor structure excluding the absorbing membrane. A dedicated reference device is fabricated for each batch of fabrication. The measured parasitic radiation Q_{ref} is subtracted from the experimental heat flows measured for all the near-field devices fabricated in the same batch to find Q_{mem}. Figure S5c shows the thermal radiation measurement results obtained from all reference devices. To consistently subtract the parasitic radiation, a fifth-order polynomial fitting with least absolute shrinkage and selection operator (LASSO) regularization is applied, as shown by the solid lines in Fig. S5c.

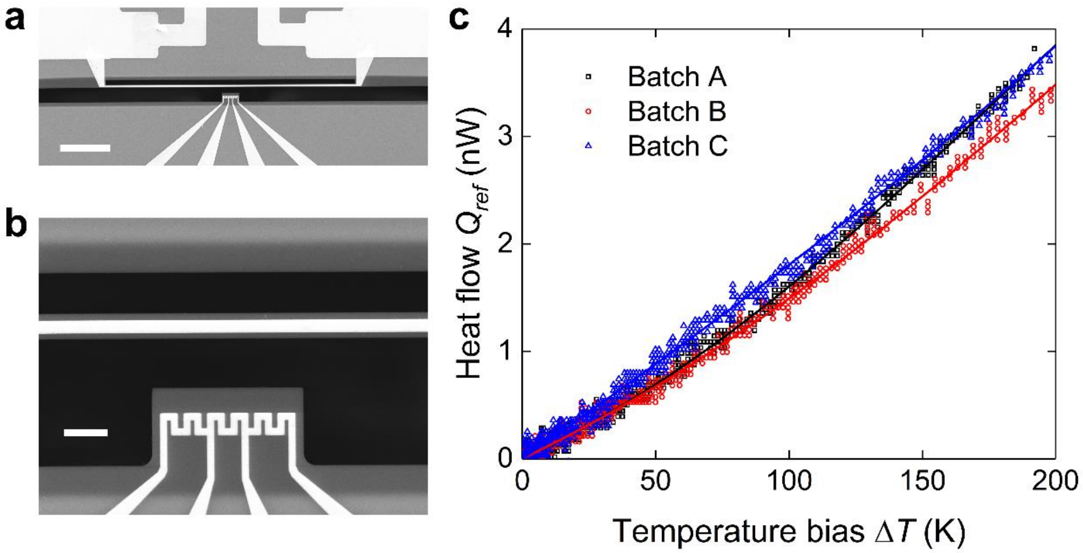

Fig. S5. Reference nanodevices. a & b, SEM images of a reference device. Scale bar: 20 μm and 2 μm, respectively. **c**, Measured radiation Q_{ref} of reference devices. Solid lines indicate fifth-order polynomial fitting with LASSO regulation.

6 Overview of thermal measurement

All the near-field thermal radiation measurements are conducted under high vacuum (10^{-3} Pa) and precise temperature control. We first calibrate the heat conductance of the sensor G_s by applying a DC current (up to 20 μA) and measuring the corresponding temperature increase through simultaneously supplying a small sinusoidal current (0.4 μA, 16Hz) and measuring the 4-wire sinusoidal voltage. Then, we measure the near-field radiation by heating up the emitting membrane using a DC current (up to ~800 μA with a 5 μA interval) and monitor the temperature increase of the sensor membrane using a Wheatstone bridge circuit. The calibration of the platinum thermometers includes temperature-resistance calibration, thermal frequency domain and noise floor (See Supplementary Information Session 7, 8, 11 for the results). Based on the platinum thermometry, we derive the temperature increases from the measured resistance variations (See Supplementary Information Session 9 for the determination of ΔT_h and ΔT_s). We collect ten data points at each DC current applied to the heater and use linear interpolation to determine the heat flow at integer temperature biases (See Supplementary Information Session 12 for details). Based on the measured heat flow, we further calculate the radiation heat conductance between the two membranes, $G_{mem} = Q_{mem}/\Delta T$.

7 Temperature-resistance calibration of PRT

The consistent temperature-resistance relation of a platinum resistance thermometer (PRT) enables the determination of the temperature by measuring the resistance. For a small temperature variation (~10K), the linear relation is accurate enough to fit between temperature and resistance. Temperature coefficient of resistance α_1 (TCR) of PRT is a measure of the linear relation, which is given by:

$$\alpha_1 = \frac{1}{R_0}\frac{dR}{dT} \tag{S1}$$

where R_0 is the resistance at a reference temperature T_0. We select 300 K as the reference temperature.
For large temperature variations (>100K), the parabolic fitting is more accurate:

$$R(T) = R_0[1 + \alpha_2(T - T_0) + \beta_2(T - T_0)^2] \tag{S2}$$

The temperature-resistance relation calibration of the PRT is performed by sweeping the ambient temperature and measuring the corresponding resistance. Figure S6 shows the measured resistances of the heater and sensor for a temperature range of 280K to 470K. During the near-field radiation measurement, the sensor undergoes a minor temperature variation and thus linear fitting around 300K is applicable to calibrate the temperature-resistance relation of the sensor. Consequently, the α_1 value of the sensor resistance R_s is 1.788×10^{-3} K^{-1}. For heater resistance R_h, parabolic fitting is applicable, which yields 1.763×10^{-3} K^{-1} for α_2 and -1.046×10^{-6} K^{-2} for β_2, respectively.

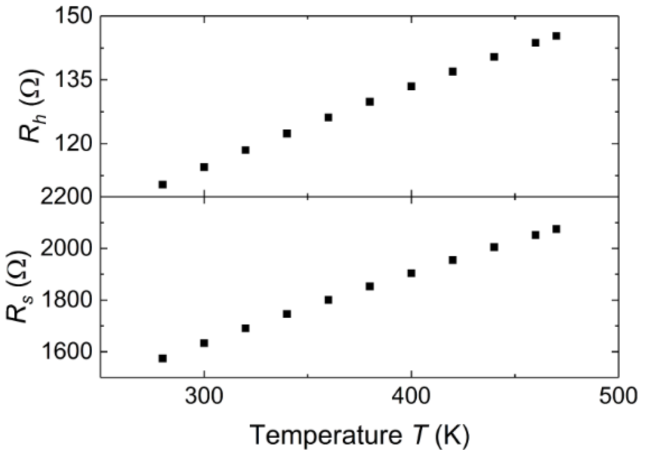

Fig. S6. Temperature-resistance relation of heater and sensor.

8 Thermal frequency domain responses of the heater & sensor

The frequency-domain thermal response is measured by applying a small sine wave current i with a large offset I_0 (large enough to induce notable temperature increase) and measure the increase of the sinusoidal voltage amplitude ΔV using a lock-in amplifier. As suggested by literature[2], the voltage increase ΔV is a function of the frequency of the sinusoidal current i:

$$\Delta V(I) = \begin{cases} 3i\Delta T(I)\dfrac{dR(I=0)}{dT}, f \ll f_0 \\ i\Delta T(I)\dfrac{dR(I=0)}{dT}, f \gg f_0 \end{cases} \tag{S3}$$

where f_0 is the thermal cutoff frequency of the heater or sensor. There is a factor-of-3 difference between the voltage increases ΔV between modulation frequencies much lower and much higher than the thermal cutoff frequency. Figure S7 shows the frequency domain responses of the sensor and heater, respectively. The thermal cutoff frequency of the heater is at ~10^4 Hz magnitude, while that of the sensor is ~10^2 Hz magnitude.

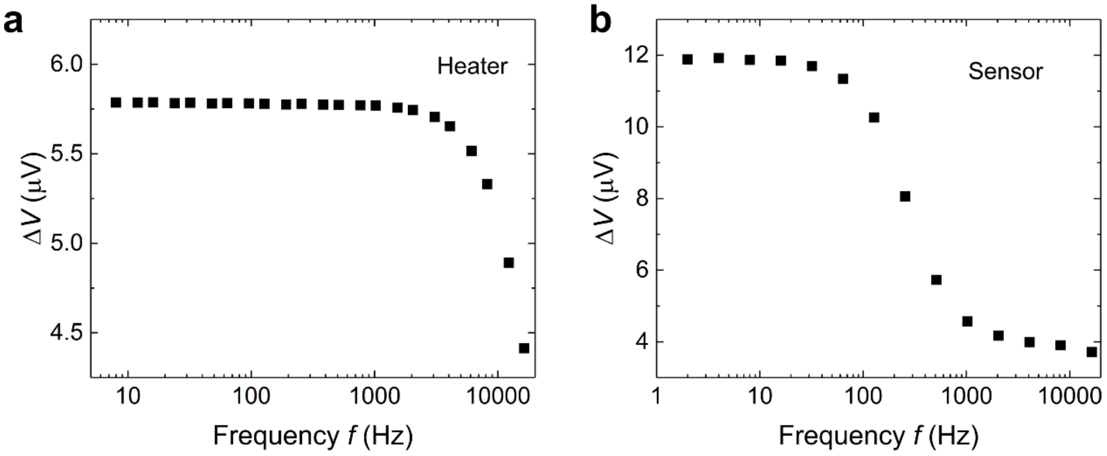

Fig. S7. Thermal frequency domain responses. a, heater, and **b**, sensor.

9 Determining temperature variations from the platinum thermometer

To determine the temperature variation of the heater, the classical four-probe connection is performed. We use the first harmonic voltage V_h to determine the temperature increase of the heating membrane ΔT_h. The frequency of the sinusoidal current, 211Hz, is much lower than the cutoff frequency of the heater, which indicates the 3 times relation at the low-frequency limit (See Section 8 Thermal frequency domain responses of the heater & sensor). To determine ΔT_h from the first harmonic voltage V_h, here, we use a modified equation of the low-frequency case in equation S3, incorporating the resistivity change induced by the low-frequency temperature modulation,

$$\Delta V_h/i = R_0\left(\alpha_2\Delta T_h + \beta_2\Delta T_h^2\right) + \frac{2R_0\Delta T_h(\alpha_2 + 2\beta_2\Delta T_h)\left(1 + \alpha_2\Delta T_h + \beta_2\Delta T_h^2\right)}{1 - \beta_2\Delta T_h^2} \tag{S4}$$

where *i* is the amplitude of the sinusoidal current.

To calibrate G_s of the sensing beam, the four-probe connection with a low frequency (16Hz) sinusoidal current i is used when applying Joule heating. The 3-time relation at the low-frequency limit is used to determine the spatial-average temperature increase of the sensor $\overline{\Delta T_{s,J}}$ due to Joule heating,

$$\overline{\Delta T_{s,J}} = \frac{\Delta R_{s,J}}{3\alpha_1 R_{s,0}} \tag{S5}$$

where $\Delta R_{s,J} = \Delta V_{s,J}/i$, and $R_{s,0}$ is the resistance of the sensor at 300K.

During the near-field radiation measurement, the Wheatstone circuit is used to determine the temperature increase of the sensor. A sinusoidal 0.01V voltage is applied to the Wheatstone bridge, and the frequency is optimized to 1531 Hz for minimizing the influence of self-heating. The spatial-average temperature increase of the sensor can be determined from the resistance change,

$$\overline{\Delta T_s} = \frac{\Delta R_s}{\alpha_1 R_{s,0}} \tag{S6}$$

10 Temperature profile along the sensor beam and calibration of *G_s*

The heat transfer along the sensor beam can be approximated by a 1D heat conduction model. We approximate the near-field heat flow as a localized heat input at the center of the beam sensor. Also, we assume the temperature at the base of the sensor equals the ambient temperature, then the heat transfer equation is,

$$-kA\frac{d^2T}{dx^2} = 0 \tag{S7a}$$

$$T(x=L) = T_{amb}, -kA\left.\frac{dT}{dx}\right|_{x=0^+} = \frac{1}{2}Q_{rad} \tag{S7b}$$

$$T(x=-L) = T_{amb}, -kA\left.\frac{dT}{dx}\right|_{x=0^-} = \frac{1}{2}Q_{rad} \tag{S7c}$$

where T_{amb} is the ambient temperature, and the x coordinate spans from -L to L. A is $(A_{Pt} + A_{SiN})$. The effective thermal conductivity $k = (k_{Pt}A_{Pt} + k_{SiN}A_{SiN})/(A_{Pt} + A_{SiN})$. By applying the temperature boundary conditions (S7b, c) to Eq. (S7a), the temperature distribution is derived as

$$T(x) = \begin{cases} -\frac{Q_{rad}}{2kA}x + \frac{Q_{rad}}{2kA}L + T_{amb}, x \geq 0 \\ \frac{Q_{rad}}{2kA}x + \frac{Q_{rad}}{2kA}L + T_{amb}, x < 0 \end{cases} \tag{S8}$$

Then, the spatial-average temperature increase of the sensor $\overline{\Delta T_s}$ is derived by integrating along the sensor,

$$\overline{\Delta T_s} = \frac{1}{2L}\int_{-L}^{L} T(x)dx - T_{amb} = \frac{Q_{rad}}{4kA}L \tag{S9}$$

Here, the temperature of the absorbing membrane T_s is equal to the temperature at the center (x = 0), and the heat conductance of the sensor G_s is,

$$G_s \equiv \frac{Q_{rad}}{\Delta T_s} = \frac{Q_{rad}}{2\overline{\Delta T_s}} = \frac{2kA}{L} \tag{S10}$$

where ΔT_s is the temperature increase of the absorbing membrane (x = 0).

To calibrate the heat conductance of the sensor beam G_s, we apply a heating current I and measure the corresponding average temperature increase $\overline{\Delta T_{s,J}}$, induced by the Joule heating power $Q_{s,J}$. The corresponding heat transfer problem can be modelled by

$$-kA\frac{d^2T}{dx^2} = \frac{Q_{s,J}}{2L} \tag{S11a}$$

$$T(x = L) = T(x = -L) = T_{amb} \tag{S11b}$$

$$Q_{s,J} = I^2R_s \tag{S11c}$$

By solving Eq. (S11) with the given boundary conditions, the temperature distribution is found as

$$T(x) = -\frac{Q_{s,J}}{4kAL}x^2 + \frac{Q_{s,J}L}{4kA} + T_{amb} \tag{S12}$$

Then the spatial-average temperature increase of the sensor due to Joule heating $\overline{\Delta T_{s,J}}$ is,

$$\overline{\Delta T_{s,J}} = \frac{1}{2L}\int_{-L}^{L} T(x)dx - T_{amb} = \frac{Q_{s,J}}{6kA}L \tag{S13}$$

Then, by substituting the kA/L in Eq. (S10) with Eq. (S13), we obtain

$$G_s = \frac{1}{3}\frac{Q_{s,J}}{\overline{\Delta T_{s,J}}} \tag{S14}$$

To test the accuracy of the 1D heat transfer model, we simulate the heat transfer in the sensor structure using finite element simulation, implemented by COMSOL Multiphysics. As shown in Fig. S8a, we first simulate the Joule heating scenario by applying a uniform heat source with 1 μW total power and then calculate the heat conductance Gs using Eq. (S14). We then simulate the near-field radiation scenario by applying a 1 μW power heat input on the 7×0.3 μm^2 lateral surface of the absorbing membrane, as shown in Fig. S8b. Figure S8c shows the temperature profile along the sensor of the two cases: radiation heat input indicates an almost linear temperature distribution while Joule heating indicates a parabolic temperature distribution. To validate the heat transfer model for the sensor, we calculate the radiation heat flow by $Q_{rad} = 2G_s\overline{\Delta T_s}$ by using the simulated temperature distribution and compare it with the real heat flow - 1 μW, as shown in Table S2. The error rate is less than 1%. To further estimate the error of thermal radiation to the ambient, we assume blackbody emissions on all the surfaces of the sensor and redo the procedure above. The error rate increases but is still less than 1%.

Table S2. Error estimation of the 1D heat transfer model for the sensor.

**Ambient radiation**	$\overline{\Delta T_s}$ **(K)**	$\overline{\Delta T_{s,J}}$ **(K)**	G_s **(nW/K)**	Q **(nW)**		**Error (%)**
				real	calculated	
**N**	8.2300	5.4998	60.610	1000	997.6	-0.24
**Y**	8.1084	5.4276	61.415	1000	995.9	-0.41

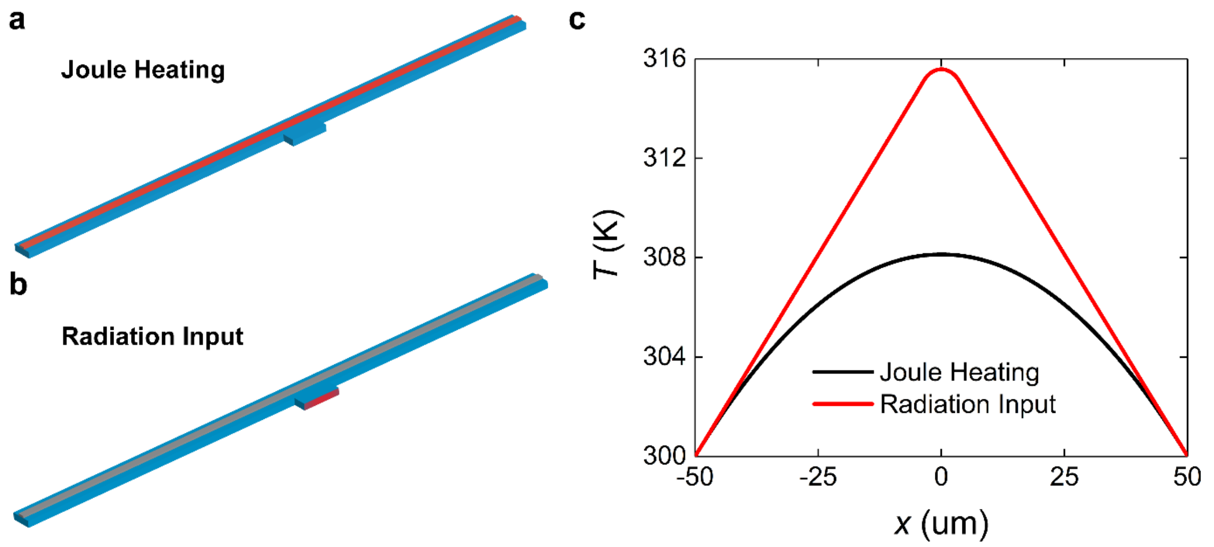

Fig. S8. Finite element simulation of heat transfer within the sensor structure. a, & **b,** the two simulation scenarios, Joule heating and radiation input, for determining the error of the 1D heat transfer model. **c,** The temperature profiles along the sensor of these two scenarios.

11 Noise floor of the measurement

To determine the noise floor of the near-field measurement, we plot the measured heat flow data for a range of small temperature bias. As shown in Fig. S9, the heat flow decreases as the temperature bias decreases from the highest temperature bias in the measurement; however, our measurement technique cannot resolve the heat flows less than ~200 pW arising when the temperature bias is below nearly 1 K. Accordingly, the noise floor is determined as ~200 pW.

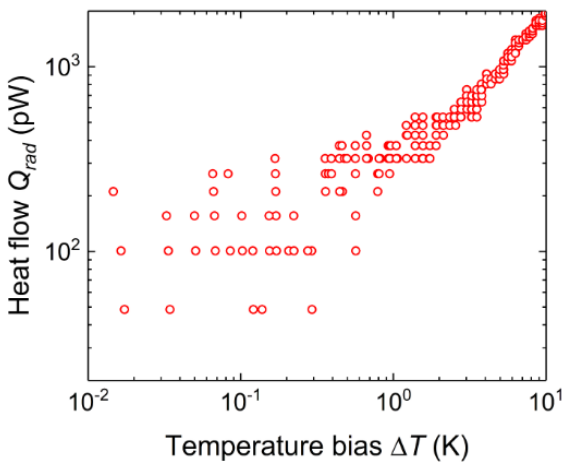

Fig. S9. Noise floor of the thermal measurement. The noise floor is ~200 pW.

12 Linear interpolations of raw data

To condense the measurement data for a given nanodevice, the raw data, e.g., black rectangles in Fig. S10, is linearly interpolated over a range of 16 K at temperature biases with 9-K increments between the neighbor data points, e.g., red filled circles in Fig. S10. Heat flow data reported in Fig. 2 and Fig. 3 in the main text follows this procedure. The maximum error arising from the interpolation is ~0.9 nW.

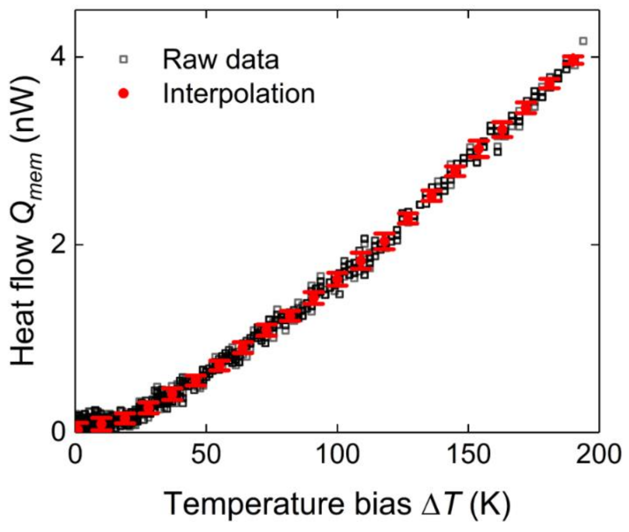

Fig. S10. Linear interpolation of the raw data. The error bar indicates the error from the linear fitting. The range of interpolation is 16K and the maximum error is ~0.9 nW.

13 Radiation heat flows of all measured devices

Figure S11 shows the radiation heat flows Q_{mem} of all measured devices and the simulated heat flow of similar gap distances.

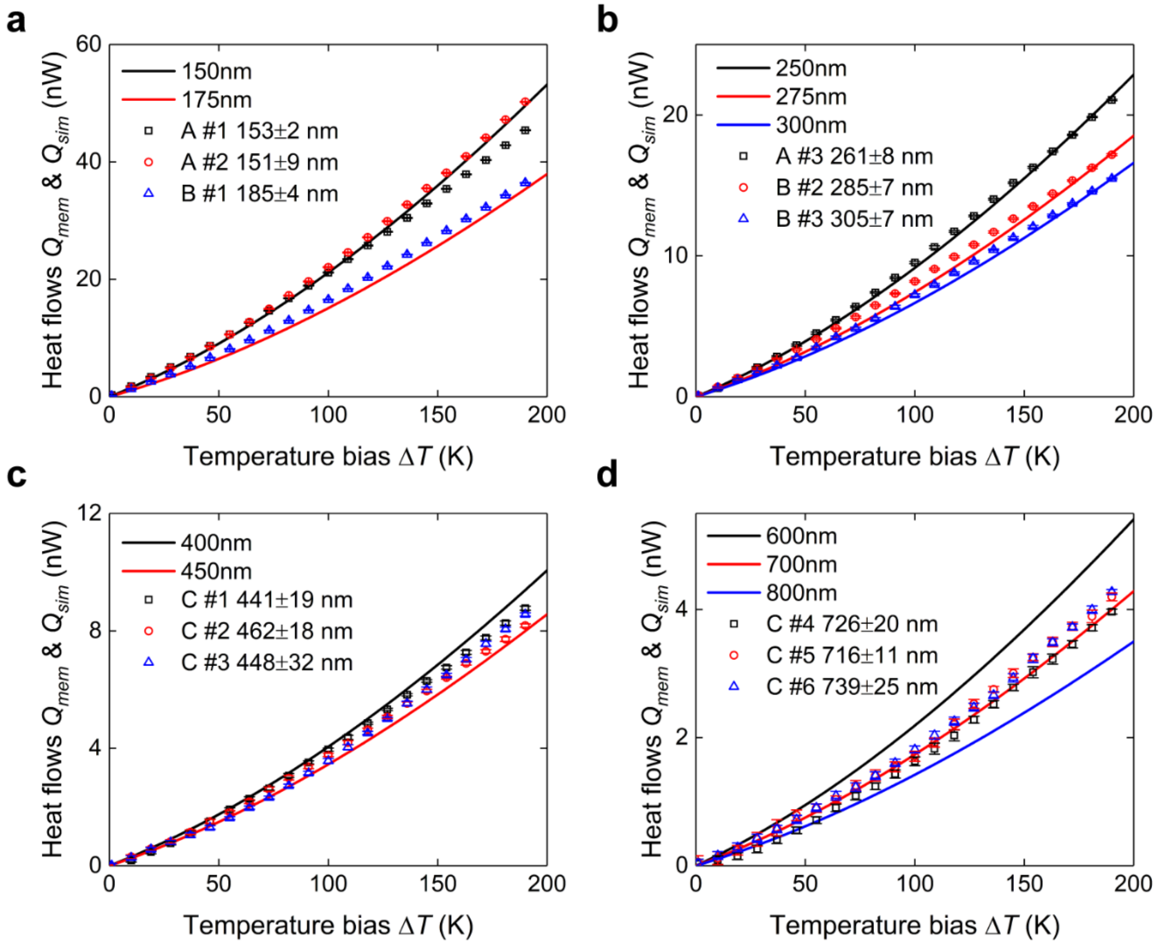

Fig. S11. Heat flow data of all measured nanodevices. Markers represent experimental data. Solid lines represent the simulation using FSC method.

14 Radiation heat conductance of all measured devices

Figure S12 shows the radiation heat conductance G_{mem} of all measured devices, which is calculated by $Q_{mem}/\Delta T$. For all gap distances, the heat conductance of radiation G_{mem} increases with a higher temperature bias. The error of G_{mem} derives from the heat flow error.

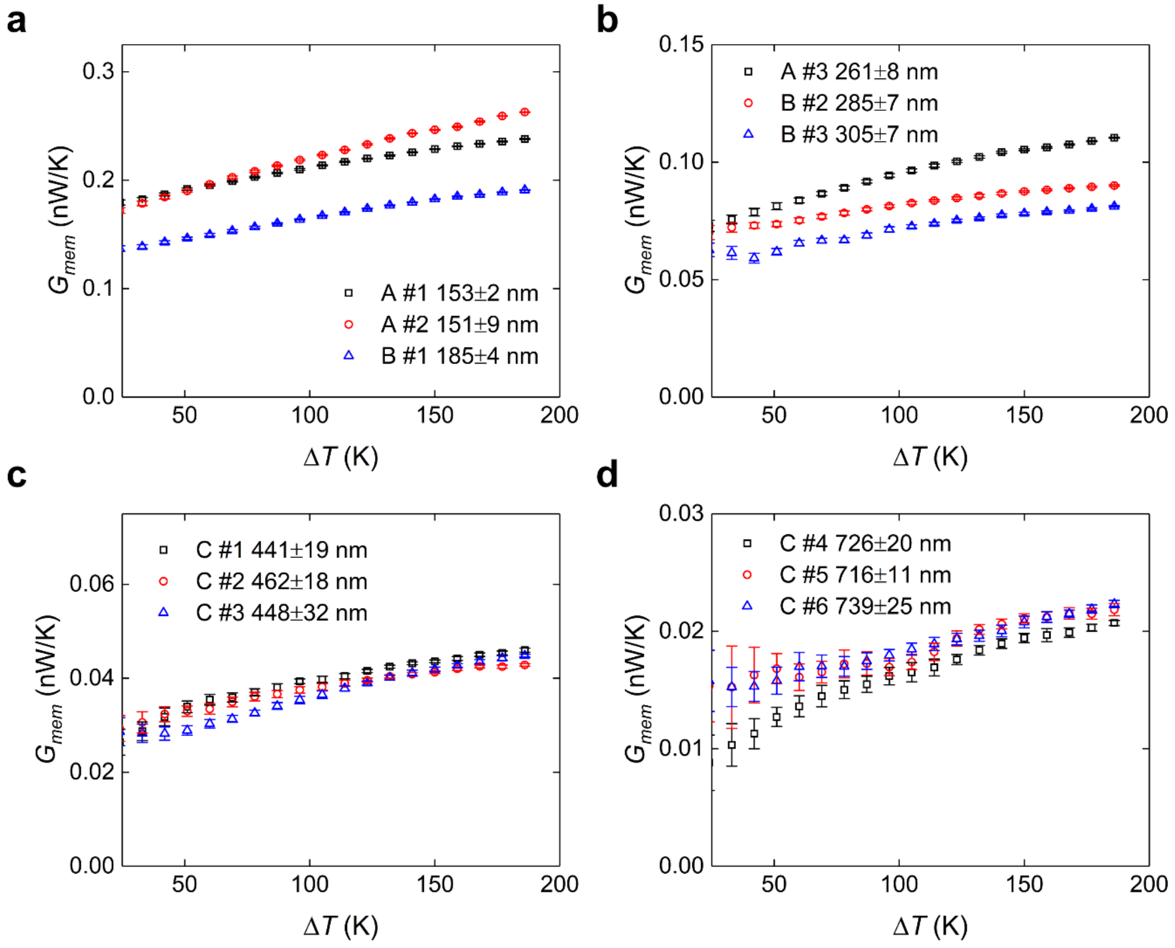

Fig. S12. Heat conductance of radiation G_{mem} of all measured nanodevices.

15 Near-field enhancements of all measured devices

Figure S13 shows the near field enhancements of all measured devices. For all gap distances, the enhancement ε decreases with higher temperature bias. The error of enhancement ε derives from two factors: the error of interpolated heat flows and the error of view factor from the deviation of gap distance measurements.

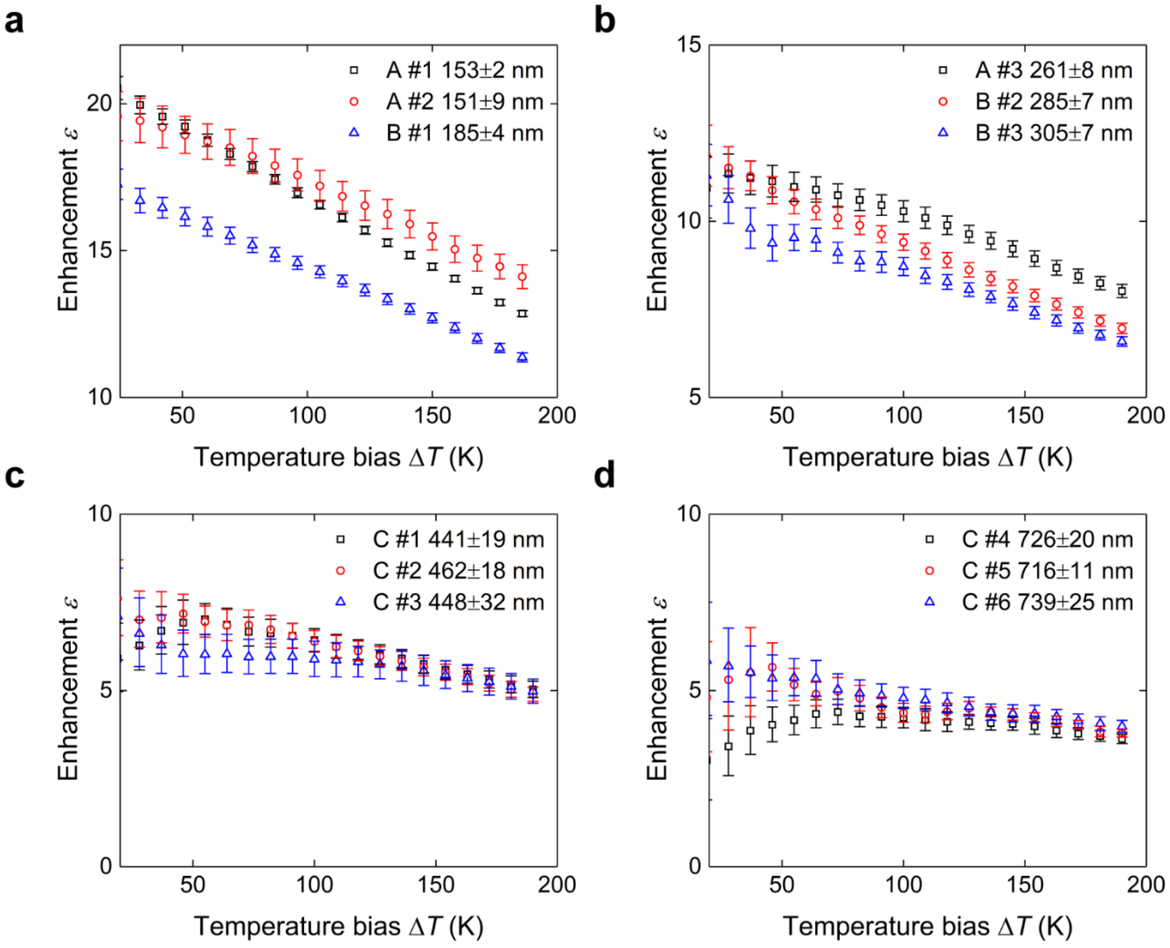

Fig. S13. Enhancement ratio ε of all measured nanodevices.

16 View factor calculation

The view factor between the exchanging surfaces is calculated by[S3]

$$F_{12} = \frac{2}{\pi XY}\left(\begin{array}{l} ln\left\{\left[\frac{(1+X^2)(1+Y^2)}{1+X^2+Y^2}\right]^{\frac{1}{2}}\right\} + X\sqrt{1+Y^2}\,tan^{-1}\frac{X}{\sqrt{1+Y^2}} \\ +Y\sqrt{1+X^2}\,tan^{-1}\frac{Y}{\sqrt{1+X^2}} - X\,tan^{-1}X - Y\,tan^{-1}Y \end{array}\right) \quad \text{(S15)}$$

where $X = \frac{t}{d}$, $Y = \frac{l}{d}$. t, l, and d are the thickness and length of the membrane, and gap distance, respectively. Figure S14 shows the view factors between two 7×0.3 μm^2 surfaces as a function of gap distance.

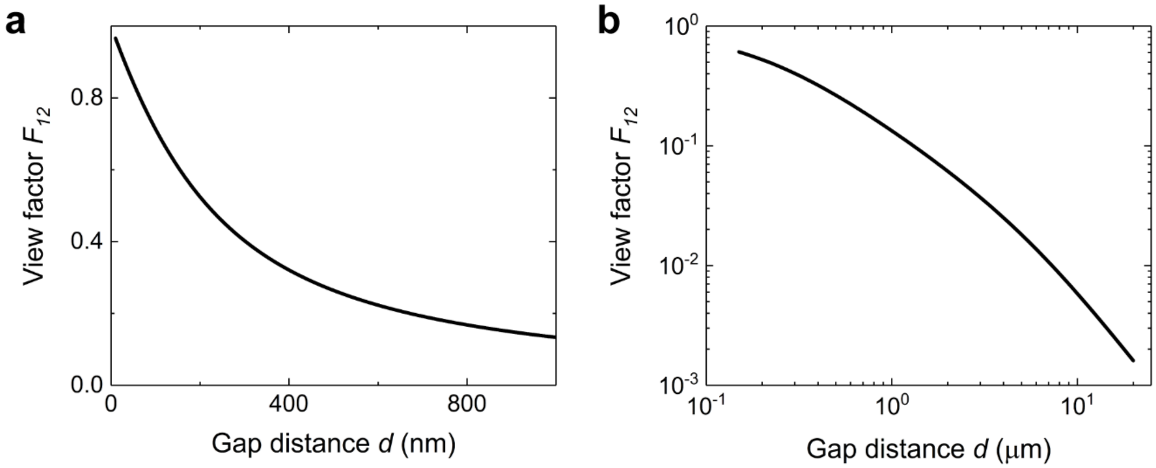

Fig. S14. View factor F_{12} between two parallel 7×0.3 μm^2 rectangles. The view factor drastically decreases as the gap distance increases from 100 nm to 1 μm **(a)**, and from 150 nm to 20 μm **(b)**.

17 Dielectric permittivity of silicon nitride

We use Maxwell-Helmholtz-Drude dispersion model[S4] to characterize the dielectric properties of the silicon nitride membranes used in our experiments, as plotted in Fig. S15.

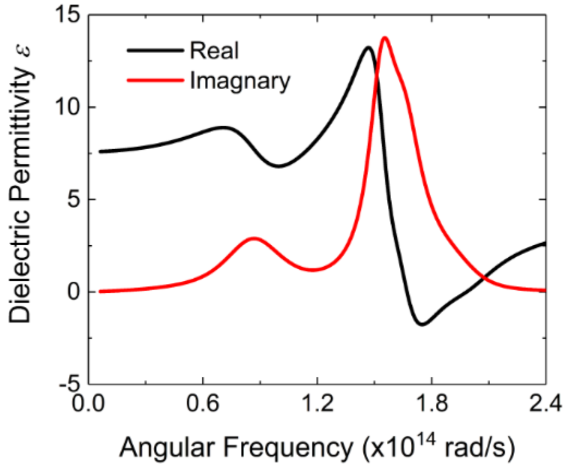

Fig. S15. Dielectric permittivity of the silicon nitride membrane.

18 Comparison with far-field super-Planckian thermal radiation

We notice that the maximum enhancement ratio we observed in our near-field measurement (~20) is less than the 100-fold enhancement reported in the far-field with similar membrane thickness[S5]. We believe such discrepancy arises because the power of blackbody radiation is much weaker for the far-field gap distance in Ref [S5] than that at the near-field gap distance in our work. Below, we quantitatively explain the discrepancy by further calculations.

We first investigate the influence of gap distance on blackbody radiation. The gap distance influences blackbody radiation by the view factor F_{12}:

$$Q_{bb} = \sigma A F_{12}(d)\left(T_h^4 - T_s^4\right) \quad \text{(S16)}$$

As shown in Fig. S14b, the view factor F_{12} highly depends on gap distance. It drops from ~0.61 to ~0.0016 as the gap distance increases from 150 nm to 20 μm. Therefore, under the same emitter and absorber temperatures, the ratio of blackbody thermal radiation at a near-field gap distance of 150 nm, $Q_{bb,NF}$, to that at a far-field distance of 20 μm, $Q_{bb,FF}$, is

$$\frac{Q_{bb,NF}}{Q_{bb,FF}} = \frac{F_{12,NF}}{F_{12,FF}} = \frac{F_{12}(150\text{nm})}{F_{12}(20\mu\text{m})} \approx 378 \quad \text{(S17)}$$

As indicated by our experiment, the near-field thermal radiation Q_{NF} is ~20 times greater than blackbody radiation at the 150 nm gap distance $Q_{bb,NF}$,

$$\frac{Q_{NF}}{Q_{bb,NF}} = 20 \tag{S18}$$

As predicted by Ref [S5], we assume the far-field thermal radiation between 300-nm-thick membranes, i.e. our membrane thickness, at 20-μm gap distance Q_{FF} is 100 times greater than blackbody thermal radiation at the given gap distance $Q_{bb,FF}$,

$$\frac{Q_{FF}}{Q_{bb,FF}} = 100 \tag{S19}$$

Now we find the near-field enhancement at 150 nm gap distance with respect to the far-field thermal radiation at 20 μm gap distance between membranes (Q_{NF}/Q_{FF}) by inserting the calculated values in Eq. (S17-19) as follows:

$$\frac{Q_{NF}}{Q_{FF}} = \frac{Q_{NF}/Q_{bb,NF}}{Q_{FF}/Q_{bb,FF}} \times \frac{Q_{bb,NF}}{Q_{bb,FF}} = \frac{20}{100} \times 378 \approx 76 \tag{S20}$$

This result indicates that our near-field thermal radiation measurement still demonstrates ~75 times greater thermal radiation than the far-field thermal radiation in the referred study. Thus, the near-field enhancement of thermal radiation still holds true.

Here, we further compare the dielectric properties used in our work with that in Ref [S5], which are very close to each other, as shown in Fig. S16. The comparison reveals that the material properties overlap well over the frequency range of interest and play no role in the difference.

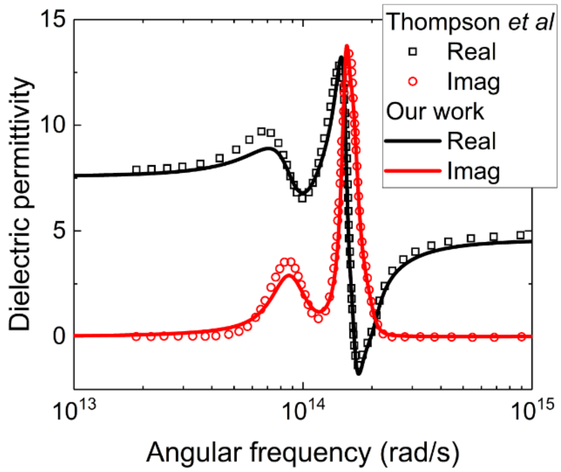

Fig. S16. Comparison of dielectric permittivity of the silicon nitride used in our work and Ref [S5].

On the other hand, we also agree that 20-fold enhancement with respect to blackbody radiation at 150-nm separation gap is modest as compared to 100-fold enhancement reported in the far-field. Based on our consideration, this may arise due to multiple reasons. First, this difference may indicate that the physical

mechanism giving rise to the enhancement in far-field measurements is different from that of the enhancement in the near-field thermal radiation. Another reason may lie in relative dimensions. In the referred study[S5], the only dimension smaller than characteristic wavelength is thickness. The small thickness (270 nm) and large width and length (80 μm and 60 μm) of the membranes may enhance thermal radiation in the far-field with strong confinement. For our study, dimensions of membranes in both lateral axes (7 μm and 2 μm) remain smaller than the characteristic wavelength (10 μm). The 100-fold enhancement in the far-field may not hold true for membranes with such small length and width. All these considerations require further investigation that is out of scope for our study, being the very first experimental study in sub-wavelength near-field thermal radiation. Certainly, these considerations point out that future studies need to be performed for better understanding of physics and the role of dimensions in thermal radiation.

19 Reflection measurement of the silicon nitride membrane

To determine the influence of temperature to the dielectric properties of silicon nitride, we measure the reflectance of suspended silicon nitride thin films for a temperature range of 323K to 498K, using Fourier Transfer Infrared Spectroscopy (FTIR). As shown in Fig. S17, the measured reflectance shows a robust reflectance profile, especially near resonance frequency (1000 cm^{-1}), and thus we assume that the dielectric properties remain constant in the temperature range considered.

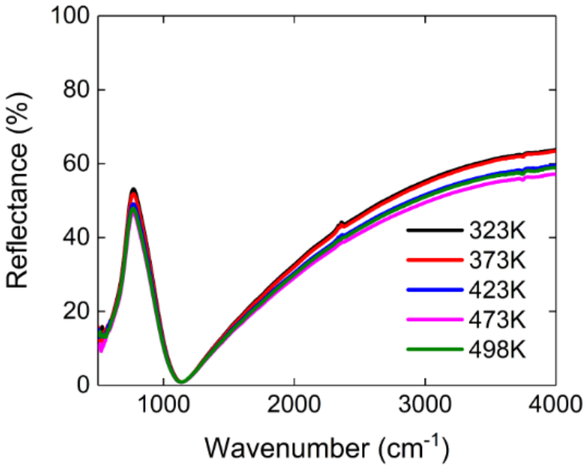

Fig. S17. FTIR reflectance measurement of the silicon nitride membrane at different temperatures.

20 Semi-infinite body calculation

We consider two planar continuous media, each at uniform hot, T_H, and cold, T_C, temperatures and separated by a vacuum gap, d, along the y axis. The heat flux from the hot (1) to cold medium (2) reads[S6]:

$$Q'' = \int \frac{d\omega}{2\pi}[\Theta(\omega,T_1) - \Theta(\omega,T_2)] \int \frac{d\kappa}{2\pi} \kappa \times \mathcal{T}_q \tag{S21}$$

$\Theta(\omega, T)$ is mean energy of Planck oscillators with frequency ω at temperature T and is given by $\hbar\omega/(e^{\hbar\omega/k_B T} - 1)$, where $\hbar$ and k_B are the reduced Planck constant and Boltzmann constant, respectively. κ denotes the wave vector component parallel to planar surface ($\kappa = k_x\hat{x} + k_z\hat{z}$). $\mathcal{T}_q$ represents energy exchange function and is given as $\frac{(1-|r_q|^2)(1-|r_q|^2)}{|1-r_q r_q e^{2ik_{y,o}d}|^2}$ for propagating, $\kappa < \omega/c$, and $\frac{4Im(r_q)Im(r_q)e^{-2Im(k_{y,o})d}}{|1-r_q r_q e^{2ik_{y,o}d}|^2}$ for evanescent, $\kappa > \omega/c$, waves. r_q stands for complex Fresnel reflection coefficient ($r_q = Re(r_q) + iIm(r_q)$ where i is imaginary unit) for q-polarization ($q = s, p$). Here, $r_s = \frac{k_{y,o}-k_{y,m}}{k_{y,o}+k_{y,m}}$ and $r_p = \frac{\varepsilon k_{y,o}-k_{y,m}}{\varepsilon k_{y,o}+k_{y,m}}$. Also, $k_{y,o} = [\omega^2/c^2 - \kappa^2]^{1/2}$, and $k_{y,m} = [\varepsilon\omega^2/c^2 - \kappa^2]^{1/2}$ are dispersion relations of polaritons in free-space and materials, respectively, where c is the speed of light, and ε is the dielectric permittivity values of the low-stress silicon nitride. To find heat transfer rate, Q_{semi}, reported in Fig. 4a, we exploit $Q_{semi} = Q'' \times A$ where A stands for the cross-section area (7×0.3 μm^2).

21 Polariton and wavelength relation

At the separation regimes of interest, κ has approximately the same magnitude of $Im(k_{y,o})$ from the dispersion relation ($\kappa \gg \omega/c$). We know that wavenumber is inversely proportional to wavelength ($\kappa = 2\pi/\lambda$ where λ is wavelength). This relation can be given for both lateral axes, separately. At relatively large distances within the interested regime, the polaritons contributing to the integration over κ in Eq. (S21) have small κ values due to $e^{-2Im(k_{y,o})d}$ term in energy exchange function in the near field. Thus, these polaritons have large wavelengths. At shorter distances, the contributing polaritons have large κ, possessing small wavelengths.

22 Electric field simulation

To quantify the impact of sub-wavelength structures on near-field radiation, we conduct finite-difference time-domain (FDTD) simulations to obtain the electric field profile near the gap. The first 250 nm-deep slot of the emitting membrane is excited based on the Wiener Chaos expansion (WCE) method[S1]. The FDTD simulation is implemented using ANSYS Lumerical. Figures 4 c & d depict the electric field profiles excited by the y-polarized dipoles.

23 The influence of height deviation

To investigate the influence of height deviation, we run our FSC simulations for the near-field thermal radiation between two membranes with height deviations at the minimum separation gap (150 nm gap) where the maximum influence of the deviation on the near-field heat transfer is expected. Our simulation results show that the reduced heat flow between the membranes with 50-nm height difference almost overlaps with that of co-planar membranes, as shown in Fig. S18. The corresponding difference of radiation heat flow is less than 1%. Thus, the influence of ~50nm height deviation to near-field thermal radiation is negligible in our study.

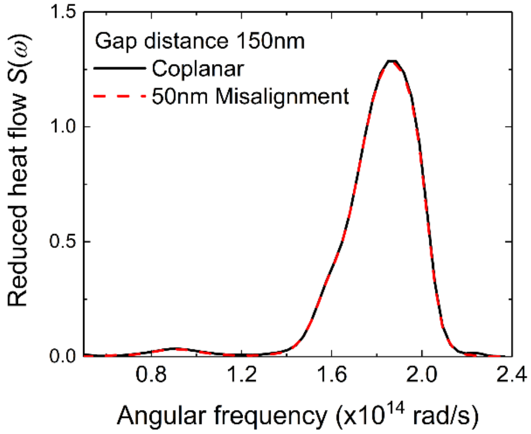

Fig. S18. The reduced heat flow between misaligned membranes with 150nm gap distance.

References

(S1) Li, J.; Liu, B.; Shen, S. Tunable Narrow-Band near-Field Thermal Emitters Based on Resonant Metamaterials. *Phys. Rev. B* **2017**, *96* (7), 075413. https://doi.org/10.1103/PhysRevB.96.075413.

(S2) Shi, L.; Li, D.; Yu, C.; Jang, W.; Kim, D.; Yao, Z.; Kim, P.; Majumdar, A. Measuring Thermal and Thermoelectric Properties of One-Dimensional Nanostructures Using a Microfabricated Device. *J. Heat Transfer* **2003**, *125* (5), 881–888. https://doi.org/10.1115/1.1597619.

(S3) Hamilton, D. C. Radiant Interchange Configuration Factors, 1949.

(S4) Cataldo, G.; Beall, J. A.; Cho, H.-M.; McAndrew, B.; Niemack, M. D.; Wollack, E. J. Infrared Dielectric Properties of Low-Stress Silicon Nitride. *Opt. Lett.* **2012**, *37* (20), 4200–4202. https://doi.org/10.1364/ol.37.004200.

(S5) Thompson, D.; Zhu, L.; Mittapally, R.; Sadat, S.; Xing, Z.; McArdle, P.; Qazilbash, M. M.; Reddy, P.; Meyhofer, E. Hundred-Fold Enhancement in Far-Field Radiative Heat Transfer over the Blackbody Limit. Nature 2018, 561 (7722), 216–221. https://doi.org/10.1038/s41586-018-0480-9.

(S6) Joulain, K.; Mulet, J.-P.; Marquier, F.; Carminati, R.; Greffet, J.-J. Surface Electromagnetic Waves Thermally Excited: Radiative Heat Transfer, Coherence Properties and Casimir Forces Revisited in the near Field. *Surf. Sci. Rep.* **2005**, *57*, 59–112. https://doi.org/10.1016/j.surfrep.2004.12.002.
